# Research advances in intramuscular fat deposition and chicken meat quality: genetics and nutrition

**DOI:** 10.1186/s40104-025-01234-5

**Published:** 2025-07-16

**Authors:** Jianlou Song, Zengpeng Lv, Yuming Guo

**Affiliations:** State Key Laboratory of Animal Nutrition and Feeding, College of Animal Science and Technology, China Agricultural University, Beijing, 100193 PR China

**Keywords:** Chicken meat quality, Embryonic nutrition, Genetic regulation, Intramuscular fat, Nutritional intervention

## Abstract

Chicken meat quality directly influences consumer acceptability and is crucial for the economic success of the poultry industry. Genetics and nutrition are key determinants of the meat quality traits in broilers. This review summarizes the research advances in this field, with a focus on the genetic and nutritional foundations that regulate intramuscular fat (IMF) deposition and meat quality in chickens over the past decade. The effects of embryonic nutrition, both maternal nutrition and in ovo feeding (IOF), on skeletal muscle development, the IMF content, and meat quality traits in broilers are also discussed. In genetics, single-cell RNA sequencing revealed that de novo lipogenesis predominantly occurs in myocytes, which is key to the formation of IMF in chicken muscle tissue. Fatty acid synthase (FASN) is the key enzyme involved in this process. This discovery has reshaped the traditional understanding of intramuscular lipid metabolism in poultry. Key genes, proteins, and pathways, such as *FASN*, *FABP4*, *PPARG*, *C/EBPα*, *SLC27A1*; LPL, APOA1, COL1A1; PPAR and ECM–receptor interactions signaling, have been identified to regulate IMF content and distribution by modulating fatty acid metabolism and adipogenesis. *LncHLFF* was innovatively found to promote ectopic IMF deposition in chickens via exosome-mediated mechanisms without affecting abdominal fat deposition. MiR-27b-3p and miR-128-3p were found to inhibit adipogenic differentiation by targeting *PPARG*, thereby affecting IMF formation. In nutrition, nutrigenomics research has shown that fructose enhances IMF deposition by activating ChREBP, providing new targets for nutritional interventions. Adjusting dietary components, including energy, protein, amino acids, fatty acids, and phytochemicals (e.g., rutin), has been shown to significantly improve meat quality in broilers. Maternal nutrition (e.g., intake of energy, amino acids, vitamins, and trace elements) and IOF (e.g., N-carbamylglutamate) have also been confirmed to significantly impact offspring meat quality, opening new avenues for improving embryonic nutrition. Based on these significant advancements, this review proposes strategies that integrate genetic and nutritional approaches. These strategies aim to modulate the differentiation fate of paraxial mesenchymal stem cells toward myogenic or adipogenic lineages and the interaction between muscle and adipose tissues. These insights would help to improve meat quality while ensuring the growth performance of broiler chickens.

**Graphical Abstract:**

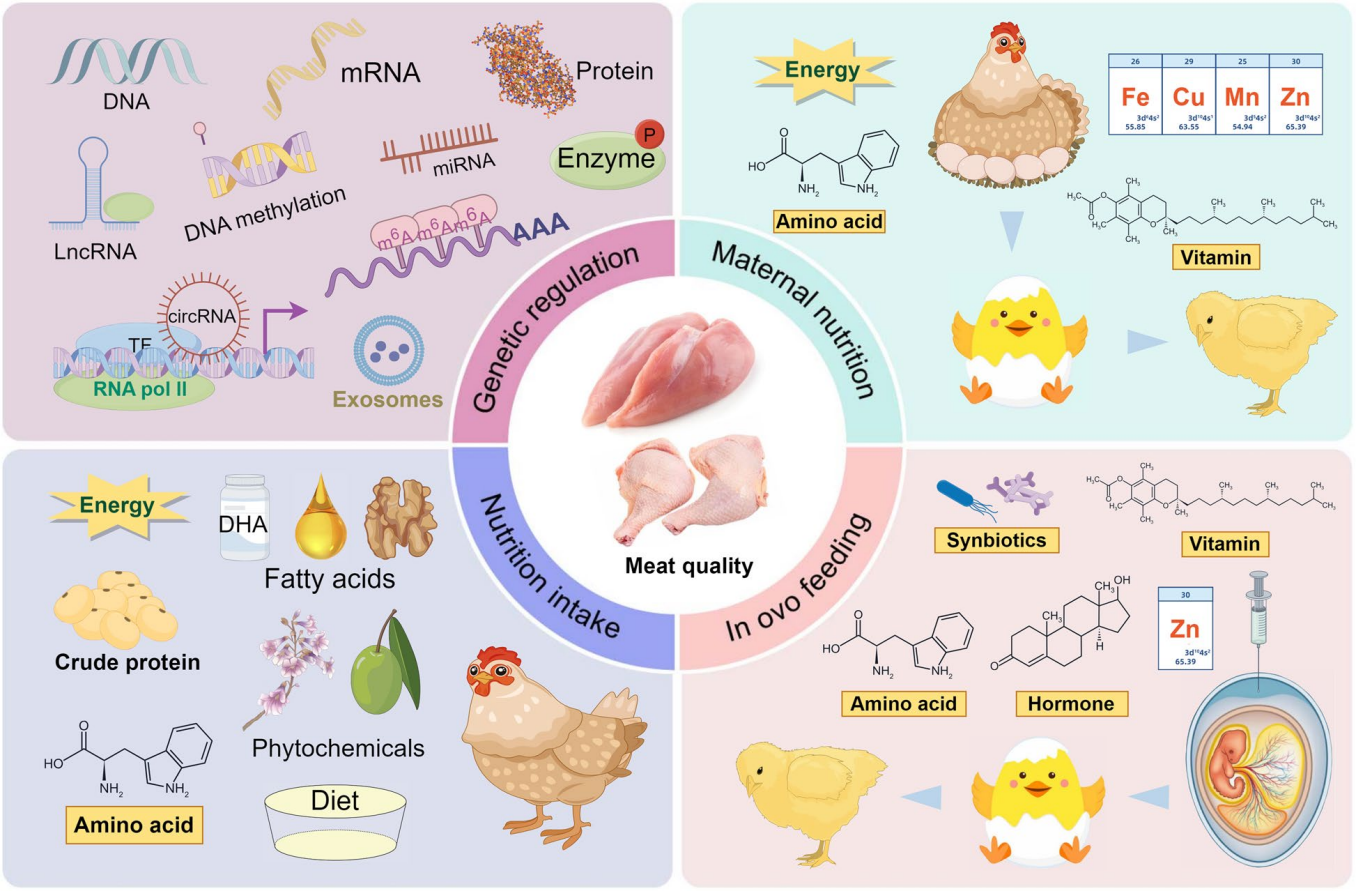

**Supplementary Information:**

The online version contains supplementary material available at 10.1186/s40104-025-01234-5.

## Introduction

As global living standards continue to improve, consumers are increasingly prioritizing the quality of meat over its quantity. However, decades of genetic selection for high growth rates and carcass yields have led to a decline in meat quality. In particular, the widespread farming of fast-growing broiler breeds has made chicken meat quality problems more prominent and attracted increasing consumer attention. In the rapid-growing broilers, an imbalance between muscle and fat development within the muscle tissue usually occurs and results in a loose texture and plain flavor. These issues fail to meet consumer demands for high-quality products that are tender, juicy, and rich in flavor [1, 2]. Therefore, there is an urgent need to improve meat quality while maintaining high meat yields.

**Fig. 1:**
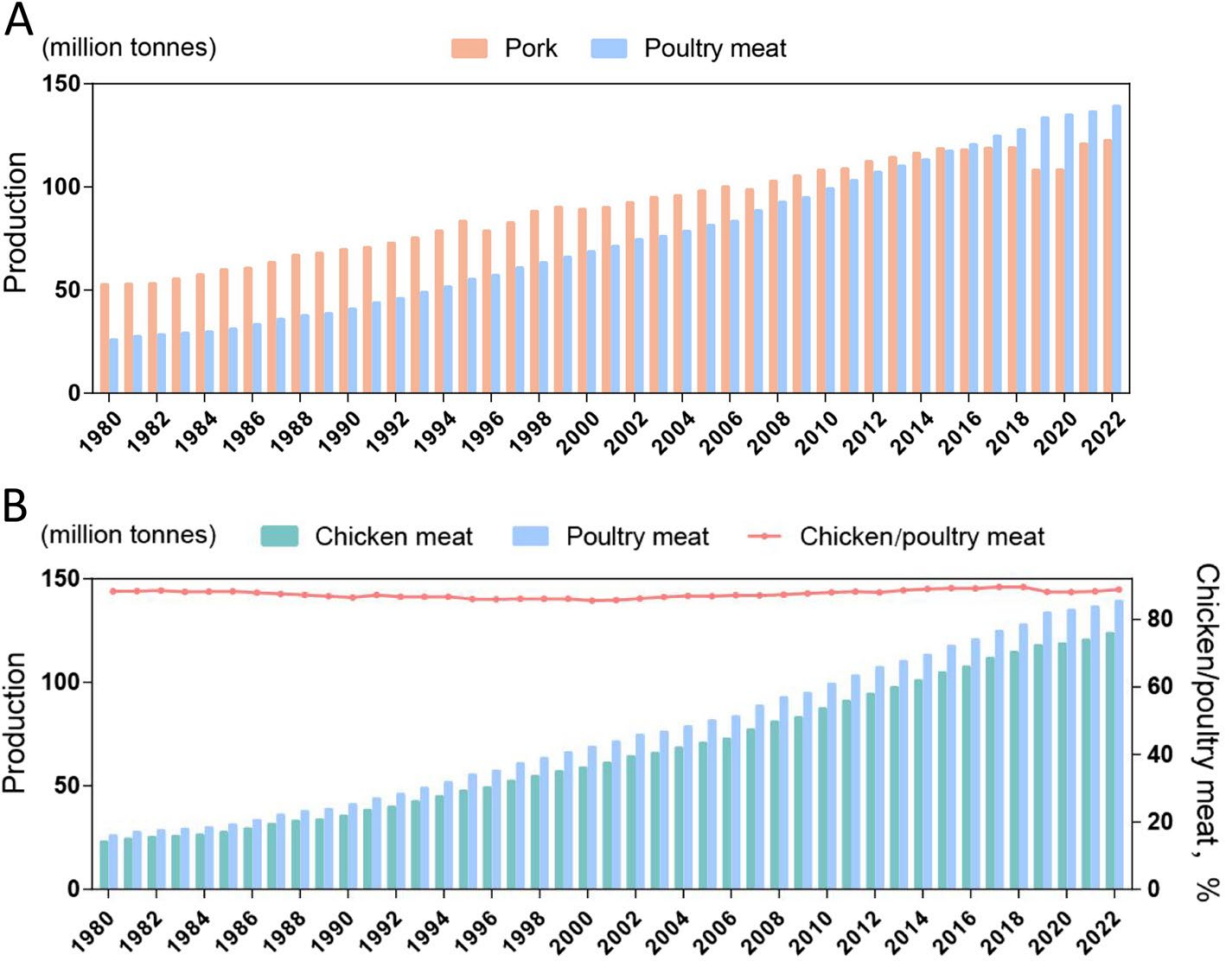
Global poultry meat and pork production from 1980 to 2022. **A** Total global poultry meat and pork production. **B** Global chicken meat production and its proportion of total poultry meat. *Source: Our World in Data* (https://ourworldindata.org/)

In the global meat consumption market, chicken meat has become one of the fastest-growing meat products due to its high nutritional value and health benefits [3]. According to the Food and Agriculture Organization (FAO), global chicken meat production has reached 139.22 million tonnes in 2022, surpassing pork to become the primary source of meat (Fig. 1). It is forecasted that by 2032, poultry consumption will account for 41% of global meat protein consumption, with particularly significant growth in chicken consumption [4]. This trend not only highlights the important position of chicken meat in the global meat market but also has posed new challenges for the sustainable development of the livestock industry. To address the growing consumer demand for high-quality chicken meat, improving meat quality, particularly by increasing the intramuscular fat (IMF) content via genetic improvement and nutritional regulation, has become a current research focus in the broiler industry [5–10], especially in China.

IMF, as a key factor affecting meat flavor, texture, tenderness, and water-holding capacity (WHC) [11], is influenced primarily by myofiber type and intramuscular adipocytes [12], which constitute the primary components of muscle and determine meat quality characteristics. In muscle, IMF exists as triglycerides (TG) within myocytes, which are particularly abundant in oxidative myofibers [13], and as lipid droplets deposited in adipocytes between myofibers or around muscle bundles. These lipid droplets form the intramuscular adipose tissue that we refer to as IMF.

Both myoblasts and intramuscular adipocytes originate from paraxial mesenchymal stem cells (MSCs), which differentiate into myogenic satellite cells and fibro-adipogenic progenitors (FAPs) during embryonic development [14–17]. Myogenic satellite cells are key cell populations for muscle regeneration and growth, whereas FAPs are the progenitors of intramuscular adipocytes [18–20]. The interaction and balance of differentiation between these cells play a decisive role in the content and distribution of IMF. Recently, FAPs have also been identified in the muscles of chicken embryos [21].

In this review, the research advances in the past decade are comprehensively summarized with respect to the genetic and nutritional factors that influence chicken meat quality. The focus is on the genetic regulatory mechanisms underlying IMF deposition as well as the impact of nutritional interventions on IMF and overall meat quality. The effects of embryonic nutrition, including maternal nutrition and in ovo feeding (IOF), on the development of skeletal muscle, IMF content, and meat quality traits in broilers are discussed. Integrative strategies that combine genetic and nutritional approaches to modulate the differentiation pathways of paraxial MSCs toward myogenic or adipogenic lineages and the interaction between muscle and adipose tissues are proposed. By providing comprehensive references and scientific insights, we aim to offer effective approaches for improving poultry meat quality. This work also supports the ongoing development of the chicken industry and provides guidance and inspiration for future research efforts.

## Genetic basis for IMF deposition in chickens

**Fig. 2:**
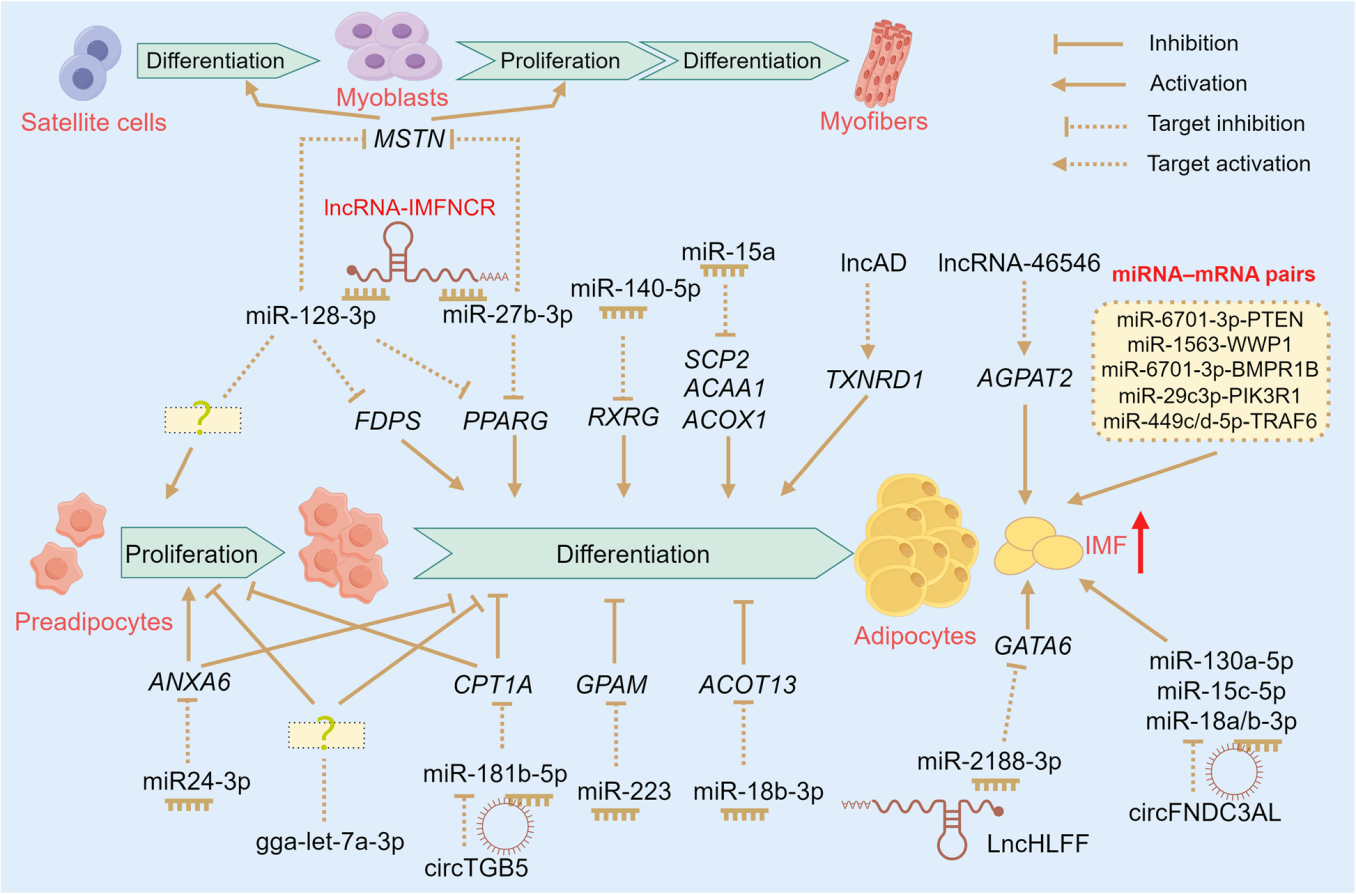
Regulatory networks of noncoding RNAs in preadipocytes and intramuscular fat (IMF) deposition. *ACAA1*: Acetyl-CoA acyltransferase 1; *ACOX1*: Acyl-CoA oxidase 1; *ACOT13*: Acyl-CoA thioesterase 13; *AGPAT2*: 1-acylglycerol-3-phosphate-O-acyltransferase 2; *ANXA6*: Annexin A6; *circ*: Circular RNA; *CPT1A*: Carnitine palmitoyl transferase 1 A; *FDPS*: Farnesyl diphosphate synthase; *GATA6*: GATA binding protein 6; *GPAM*: Glycerol‑3‑phosphate acyltransferase; lncRNA/lnc: Long noncoding RNA; miR: MicroRNA; *MSTN*: Myostatin; *PPARG*: Peroxisome proliferator activated receptor γ; *RXRG*: Retinoid X receptor γ; *SCP2*: Sterol carrier protein 2; *TXNRD1*: Thioredoxin reductase 1. The red upward arrow represents an increase. The question mark within the dashed brown box represents the target gene that has not yet been identified. Created by FigDraw (https://www.figdraw.com)

**Fig. 3:**
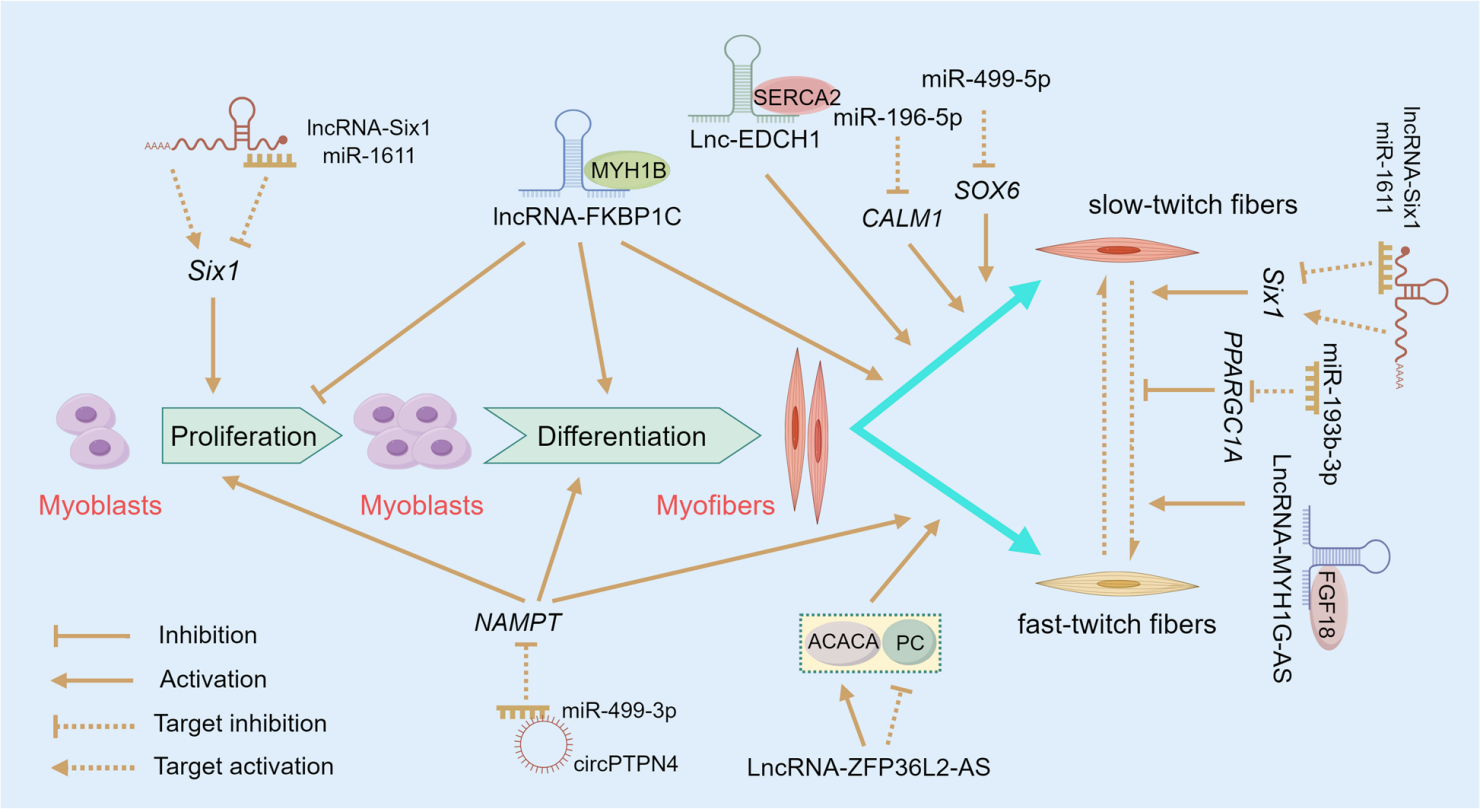
Regulatory networks of noncoding RNAs in the regulation of myofiber type profiles. ACACA: Acetyl-CoA carboxylase alpha; *CALM1*: Calmodulin 1; *circPTPN4*: Circular RNA *PTPN4*; FGF18: Fibroblast growth factor 18; lncRNA/lnc: Long noncoding RNA; miR: MicroRNA; MYH1B: Myosin heavy chain 1B; *NAMPT*: Nicotinamide phosphoribosyltransferase; PC: Pyruvate carboxylase; *PPARGC1A*: Peroxisome proliferator-activated receptor gamma coactivator 1-alpha; SERCA2: Sarcoplasmic/endoplasmic reticulum Ca^2+^ transporting 2; *SOX6*: SRY-box transcription factor 6; *Six1*: SIX homeobox 1. Created by FigDraw (https://www.figdraw.com)

IMF content, as a quantitative trait, directly responds to genetic selection and can be effectively increased through appropriate genetic selection [22–25]. Genetic studies have focused primarily on revealing the molecular regulatory mechanisms underlying IMF deposition and identifying key molecular markers and targets through comparative analysis, as shown in Table 1 and Figs. 2 and 3. This includes the expression of genes affecting IMF deposition-related signaling pathways and metabolic processes, as well as genetic variations that regulate the expression and functions of these genes, such as single nucleotide polymorphisms (SNPs), epigenetic modifications, and noncoding RNAs. These studies provide a theoretical foundation for enhancing the IMF content and improving chicken meat quality.

**Table 1:** Key pathways and genes involved in the regulation of chicken IMF deposition^a^

Perspectives	Chicken breeds	Tissues^b^	Pathways^c^	Key genes or proteins^d^	References
Interspecific variations					
	Beijing-you vs. Jingxing	Breast muscles	—	***FABP4***	[26]
	Beijing-you	Thigh and breast muscles	—	SNPs in ***FABP4*** and ***H-FABP* (*FABP3*)**	[28]
	Beijing-you vs. Arbor Acres	Breast muscles	**MAPK**, ErbB, **PPAR,** tight junction, **ECM–receptor interactions**, **focal adhesion**, and **actin cytoskeleton regulation**	*HMGCLL1*, ***THBS1***, *UCP3*, and SNX4	[29]
	Beijing-you vs. Arbor Acres	Breast muscles (at hatching)	**TGF-β**, **PPAR**, Hedgehog, and cytokine–cytokine receptor interaction signaling pathways	*ACAT1*, *CPT1A*, ***CPT2***, *DAK*, *ACOX2*, *ACOX3*, *APOO*, *FUT9*, *GCNT1*, and *B4GALT3*	[30]
	Wenchang vs. White Rock	Breast muscles	CPT1 A-mediated **fatty acid oxidation**	***SLC27A1* (*FATP1*)**	[31]
	Zhuanghe Dagu vs. Arbor Acres	Pectorales and crureus	**ECM‒receptor interactions**	*EHHADH*, *TECRL*, *NDUFAB1*, *PCCB*, and *HIBCH*	[32]
	Baicheng-you and Sanhuang	Chest and leg muscles	—	C12315T mutation in ***LPL*** (XM_015280414.2)	[33]
	Lushi vs. Arbor Acres	Breast muscles	—	*HMGCR*	[34]
	Xueshan vs. Ross 308	Pectoralis major	**mTOR**, collagen binding, and **lipid metabolism**	SLC7A5 at Ser21, MRC2 at Ser1359 and CRAT at Ser341, AUP1 at Ser377	[35]
	Yufen vs. Arbor Acres	Breast muscle	**Lipid** and **steroid** biosynthesis and metabolism pathway	*PIGO*, *PEMT*, *DHCR7*, *TMEM38B, and DHDH*	[36]
	Gushi-Anka F2 (GWAS) Gushi vs. Arbor Acres	Breast muscles	ERK/**MAPK** pathway	*DNAJC27*, *FKBP1B*, ***G3BP1***, and *SCARA5*	[37]
	Gushi	Breast muscles	—	***G3BP1***	[38]
Individual variations					
	Jingxing-yellow	Breast muscles (HTG vs. LTG)	**PPAR** and **steroid biosynthesis pathway**	***ADIPOQ***, ***CD36***, ***SCD***, ***FABP4***, *FABP5*, ***LPL***, ***PLIN1***, *CIDEC*, and ***PPARG***; *DHCR24*, *LSS*, *MSMO1*, *NSDHL*, and *CH25H*	[39]
	Jingxing-yellow	Breast muscles	**De novo lipogenesis**	*SLC16A7*	[40]
	Guizhou-yellow	Breast muscles	**Focal adhesion**, **ECM‒receptor interactions, cell adhesion molecules**, **actin cytoskeleton regulation**, and **PPAR**	*CAPN2*, ***COL1A1***, *COL1A2*, ***COL6A1***, *COL6A2*, and *COL6A3*	[41]
	Huangshan Black	Thigh muscles	**Lipid, steroid**, and **fatty acid biosynthetic** and **metabolic** pathways	***FABP4***, ***G0S2***, ***PLIN1***, ***SCD****, FABP1*, *SLC1A6*, *SLC45A3*, *ACSBG1*, *LY86*, *ST8SIA5*, *SNAI2*, *HPGD*, *EDN2*, and ***THRSP***	[42]
	Huangshan Black	Thigh muscles	**PPAR**	***G0S2***, ***SCD***, and *PNPLA2*	[43]
	Wuliang Mountain Black-bone	Breast muscles	—	***C/EBPα*** and its SNP (g.552G > A)	[44]
	Gushi-Anka F2	Breast muscles	—	*SCT2*	[45]
Tissue variations					
	Beijing-you	Breast muscles vs. leg muscles	**Focal adhesion**, **ECM‒receptor interactions**, and **PPARγ** signaling pathways	***LPL***, ***FABP4***, ***THRSP***, ***FABP3***, and ***CPT2***	[46]
	Wenchang	Breast muscles vs. abdominal adipose tissues	Pyruvate and citric acid metabolism	*GAPDH*, *LDHA*, *GPX1*, *GBE1*	[47]
	Gushi	Breast muscles vs. abdominal adipose tissues	**Glycerophospholipids** (IMF) Triacylglycerols (AF)	*TMEM164* (IMF) *ZNF488* (AF)	[48]
Developmental stages					
	Beijing-you	Breast muscles (1 d, 56 d, 98 d, and 140 d)	—	**APOA1**, **HSPB1**	[49]
	Beijing-you and Cobb	Breast muscles (ED12, ED17, 1 d, and 14 d)	Protein processing and **PPAR** signaling pathways	Rate-limiting enzymes for fatty acid β-oxidation (ACADL, ACAD9, HADHA and HADHB)	[50]
	Beijing-you	Breast muscles (3 to18 weeks)	Energy metabolism	*ACOT9*, *CETP*, *LPIN1*, *DGAT2*, *RBP7*, *FBP1*, and *PHKA1*	[51]
	Beijing-you	Thigh muscles (150 d, 300 d, and 450 d)	**PPAR**, **MAPK**, **focal adhesion**, and **ECM–receptor interactions**	APOC3, **APOA1**, STMN1, **HSPB1**, PAK2, TGF-β, CASP3, COL1A2, COL4A1, LAMB4, and **THBS1**	[52]
	Jingxing-yellow	Breast muscles and AF (ED12 to 180 d)	**Fatty acid metabolism**, **PPAR**, Cell cycle and **cell adhesion molecules**	*ENSGALG00000041996, L3MBTL1*, *TNIP1*, *HAT1*,* BEND6*	[53]
	Jingxing-yellow	Breast muscles (7, 35, 63, 91, and 119 d)	Adipocytokine, **MAPK**, **mTOR**, FoxO, and **TGF-β** signaling pathways	*RPS6KB1*, *BRCA1*, *CDK1*, *RPS3*, *PPARGC1 A*, *ACSL1*, NDUFAB1, *NDUFA9*, *ATP5B*, and *PRKAG2*	[55]
Gender and Rearing conditions					
	Lueyang black-boned	Leg muscles	**PPAR**	*ANGPTL4*, ***CD36***, ***FATP1***, *FATP4*, and ***PLIN2***	[56]
	Daheng	Breast muscles	**Fatty acid degradation**, **Fatty acid biosynthesis**, and Glycolysis/Gluconeogenesis, and **PPAR**	***PLIN2***	[59]
Cellular levels and high-throughput sequencing					
	Jingxing-yellow	Breast muscles	**Wnt**, **PPAR, focal adhesion**, **ECM–receptor interaction**, apoptosis, and AGE-RAGE signaling pathways	***APOA1***, ***COL1A1***, and*** ADIPOQ***	[64]
	Jingxing-yellow Qingyuan partridge Wenchang E line of Jinling huang	Breast muscles	**De novo lipogenesis, fatty acid biosynthesis** and **oxidation** pathway	*FASN* and its upstream mutation (rs315349829)	[65]
	Beijing-you	Breast muscles	**ECM‒receptor interactions**	*TIMP2*	[68]
	Arbor Acres	Embryonic leg muscle	—	*IGFBP7* and ***C/EBPα***	[69]
Epigenetic modification					
	Gushi	Pectoral muscles	**Wnt**, Jak-STAT, **ECM‒receptor interactions**, and **focal adhesion** signaling pathway	Hypermethylation of the promoters at *ABCA1*, ***COL6A1***, and *GSTT1L*	[70]
	Jingxing-yellow	Breast muscles	**Glycerophospholipid** metabolism, **fatty acid catabolism**, and **PPARγ** signaling pathways	*PLA2G4F*	[71]
	Jingyuan	Breast muscles	m^6^A-induced ferroptosis pathway	*LMOD2*	[72]
	Jingyuan	Breast muscles	—	*CUBN*, *MEGF10*, *BOP1*, and *BMPR2*	[73]
	Jingyuan	Breast muscles vs. leg muscles	Amino acid biosynthesis, peroxisome, **fatty acid biosynthesis**, **fatty acid elongation**, **cell adhesion molecules**	*ECH1*, *BCAT1*, *CYP1B1*	[74]

^a^The bold font highlights candidate genes and pathways linked to intramuscular fat (IMF) deposition regulation identified in more than two studies

^b^*AF* abdominal fat, *ED* Embryonic day, *HTG* High triglyceride, *LTG* Low triglyceride

^c^*AGE-RAGE* Advanced glycation end products–receptor for advanced glycation end products signaling pathway, *CPT1A* Carnitine palmitoyl transferase I A, *ErbB* Erythroblastic leukemia viral ongene homolog, *ECM‒receptor interactions* Extracellular matrix receptor interactions, *ERK* Extracellular regulated protein kinases pathway, *FoxO* Forkhead box, sub-group O signaling pathway, *Jak-STAT* Janus kinase-signal transducer and activator of transcription, *m*^6^A N^6^-methyladenosine modification of RNA, *MAPK* Mitogen-activated protein kinase, *mTOR* Mammalian target of rapamycin (mTOR) signaling pathway, *PPAR/PPARγ* Peroxisome proliferator-activated receptor (PPAR) and its subtype PPARγ signaling pathway, *TGF-β* Transforming growth factor-beta, *Wnt* Wingless and Int signaling pathway; —: the relevant pathway was not highlighted in the study

^d^Annotations of key genes or proteins in Table 1 can be found in Supplementary Material 1

### Interspecific IMF deposition variations

Slow-growing indigenous Chinese chicken breeds are more popular among Chinese people because of their higher IMF content and superior meat quality. Understanding the characteristics of IMF deposition across different breeds is essential for elucidating the molecular mechanisms of IMF formation (Table 1). For example, Beijing-you chickens, an indigenous Chinese breed renowned for its higher IMF content, were found to have significantly higher expression levels of adipocyte fatty acid binding protein 4 (*FABP4*) compared to Jingxing-yellow chickens, a breed artificially developed in China [26]. The *FABP* family, which encodes cytoplasmic proteins, has long been recognized as playing a major role in glucose and lipid metabolic functions [27]. An association analysis indicated that the IMF content of males was significantly influenced by the SNPs in the *FABP4* and heart-type fatty acid binding protein (*H-FABP*) genes of Beijing-you chickens. *H-FABP* is also known as fatty acid binding protein 3 (*FABP3*). Chickens with the BB genotype at the *FABP4* gene were detected to have a higher IMF content than those with the AA or AB genotypes, and individuals with the DD or CD genotypes at the *H-FABP* gene had a significantly higher IMF content than those with the CC genotype [28]. These findings highlight the role of the *FABP* gene family in the positive regulation of chicken IMF and suggest that the identified SNPs can be utilized to select breeds with greater genetic potential for IMF deposition.

A transcriptome analysis of the breast muscle revealed potential candidate genes associated with IMF deposition between Beijing-you chickens and Arbor Acres broilers. These genes include 3-hydroxymethyl-3-methylglutaryl-CoA lyase like 1 (*HMGCLL1*), thrombospondin 1 (*THBS1*), uncoupling protein 3 (*UCP3*), enoyl-CoA hydratase and 3-hydroxyacyl CoA dehydrogenase (*EHHADH*), and sorting nexin 4 (*SNX4*). Pathways affecting lipid metabolism and cell junctions, such as mitogen-activated protein kinase (MAPK) signaling, peroxisome proliferator-activated receptor (PPAR) signaling, tight junction, extracellular matrix (ECM)–receptor interactions, focal adhesion, and regulation of the actin cytoskeleton, were suggested to potentially contribute to IMF deposition [29]. At hatching, the difference in the IMF content of the breast muscles between Arbor Acres broilers and Beijing-you chickens was found to be related to the additional energy provided by the yolk sac. Comparative analysis revealed that this energy was transported and deposited as IMF in the pectoralis major muscle of Arbor Acres broilers. The transforming growth factor-β (TGF)-β, PPAR, Hedgehog, and cytokine‒cytokine receptor interaction signaling pathways, were found to played crucial roles in this process [30].

Potential mechanisms underlying the variations in IMF deposition among different chicken breeds have also been revealed in several studies on other indigenous Chinese chicken breeds. For example, in Wenchang chickens, the downregulation of solute carrier family 27 member 1 (*SLC27A1*) was shown to promote the IMF deposition by reducing carnitine palmitoyl transferase 1 A (CPT1A)-mediated fatty acid oxidation. *SLC27A1* is also known as fatty acid transport protein 1 (*FATP1*) [31]. In Zhuanghe Dagu chickens, ECM–receptor interactions pathway was shown to enhance IMF deposition by affecting the metabolism of intramuscular adipocytes [32]. In Baicheng-you chickens and Sanhuang chickens, the synonymous mutation C12315T in the lipoprotein lipase (*LPL*) gene (XM_015280414.2) was reported to have a significant positive correlation with IMF content [33]. Between Lushi and Arbor Acres chickens, 3-hydroxy-3-methylglutaryl-CoA reductase (*HMGCR*) was suggested as a potential biomarker for IMF deposition in breast muscle through comparative analysis. Its expression was indicated to be regulated by topologically associating domain (TAD) boundary sliding [34]. Between Xueshan and Ross 308 chickens, several phosphorylation sites of proteins were identified by phosphoproteomic analysis, such as large neutral amino acids transporter small subunit 1 (SLC7A5) at Ser21, C-type mannose receptor 2 (MRC2) at Ser1359, carn-acyltransf domain-containing protein (CRAT) at Ser341, and CUE domain-containing protein (AUP1) at Ser377. These phosphoproteins were thought to positively affect protein and IMF deposition by being involved in the mammalian target of rapamycin (mTOR) signaling pathway, collagen binding, and lipid metabolism [35]. In Yufen indigenous chickens, the limited breed-specific up-regulated genes, including phosphatidylinositol glycan anchor biosynthesis class O (*PIGO*), phosphatidylethanolamine N-methyltransferase (*PEMT*), 7-dehydrocholesterol reductase (*DHCR7*), transmembrane protein 38B (*TMEM38B*), and dihydrodiol dehydrogenase (*DHDH*), were discovered through a dynamic analysis of three-dimensional chromatin architecture. These genes were suggested to contribute to IMF deposition via the metabolic pathways of phospholipid and steroid biosynthesis and metabolism [36]. Among Gushi-Anka F2, Gushi, and Arbor Acres chickens, four shared genes associated with adipocyte formation—DnaJ heat shock protein family (Hsp40) member C27 (*DNAJC27*), FKBP prolyl isomerase 1B (*FKBP1B*), G3BP stress granule assembly factor 1 (*G3BP1*), and scavenger receptor class A member 5 (*SCARA5*)—were detected as candidate genes influencing IMF traits through the integrated analysis of genome-wide association studies (GWAS) and transcriptome analysis [37]. Among them, *G3BP1* was demonstrated to promote the proliferation and differentiation of intramuscular preadipocytes, and its specific mutation site, 13588667G > A, was found to be negatively correlated with IMF deposition [38]. These findings underscore the complex interplay of gene expression, metabolic pathways, and genetic variations in shaping the IMF content across different chicken breeds. Distinct genetic mechanisms of IMF deposition are found in fast-growing broiler breeds and Chinese indigenous chickens.

### Intraspecific IMF deposition variations

Genetic investigations into intraspecific IMF deposition within chicken breeds have focused predominantly on evaluating individual variability across specific muscles, analyzing fat content across different tissues, monitoring changes in IMF content throughout developmental stages, and examining the influence of rearing conditions and gender on IMF levels.

#### Individual variations in IMF deposition

In Jingxing-yellow chickens, comparative transcriptome analysis revealed that the PPAR signaling pathway and the steroid biosynthesis pathway play important roles in the regulation of IMF deposition (Table 1). Genes associated with adipogenesis and lipogenesis, including adiponectin, C1Q, and collagen domain containing (*ADIPOQ*), cluster of differentiation 36 (*CD36*), *FABP4*, fatty acid binding protein 5 (*FABP5*), *LPL*, stearoyl-CoA desaturase (*SCD*), perilipin 1 (*PLIN1*), cell death-inducing DNA fragmentation factor-like effector C (*CIDEC*), and peroxisome proliferator activated receptor γ (*PPARG*), as well as genes related to steroid biosynthesis, including 24-dehydrocholesterol reductase (*DHCR24*), lanosterol synthase (*LSS*), methylsterol monooxygenase 1 (*MSMO1*), NAD(P)-dependent steroid dehydrogenase-like (*NSDHL*), and cholesterol 25-hydroxylase (*CH25H*), were shown to be associated with high TG content in breast muscles [39]. A GWAS analysis identified the solute carrier family 16 member 7 gene (*SLC16A7*) as a key candidate gene associated with muscle TG content. Subsequent experiments demonstrated that *SLC16A7* promotes TG deposition in chicken myocytes by regulating de novo lipogenesis (DNL) [40]. In Guizhou-yellow chickens, a local breed in China, comparative transcriptome analysis showed that pathways such as focal adhesion, ECM‒receptor interactions, cell adhesion molecules, actin cytoskeleton regulation, and the PPAR signaling pathway may be involved in IMF deposition in the breast. Genes such as calpain 2 (*CAPN2*), collagen type I alpha 1 chain (*COL1A1*), collagen type I alpha 2 chain (*COL1A2*), collagen type VI alpha 1 chain (*COL6A1*), collagen type VI alpha 2 chain (*COL6A2*), and collagen type VI alpha 3 chain (*COL6A3*) were identified as key genes [41]. In Huangshan Black chickens (an indigenous Chinese breed), it was reported that genes, including *FABP4*, G0/G1 switch 2 (*G0S2*), *PLIN1*, *SCD*, fatty acid binding protein 1 (*FABP1*), solute carrier family 1 member 6 (*SLC1A6*), solute carrier family 45 member 3 (*SLC45A3*), acyl-CoA synthetase bubblegum family member 1 (*ACSBG1*), lymphocyte antigen 86 (*LY86*), ST8 alpha-N-acetyl-neuraminide alpha-2,8-sialyltransferase 5 (*ST8SIA5*), snail family transcriptional repressor 2 (*SNAI2*), 15-hydroxyprostaglandin dehydrogenase (*HPGD*), endothelin 2 (*EDN2*), and thyroid-hormone responsive protein (*THRSP*), were‌ differentially expressed in thigh muscles with extremely different IMF contents. These genes were found to be significantly enriched in lipid, steroid, and fatty acid biosynthetic and metabolic pathways [42]. Further experiments confirmed that *G0S2* can promote the differentiation of intramuscular preadipocytes and IMF deposition by regulating *SCD* and its downstream gene, patatin-like phospholipase domain containing 2 (*PNPLA2*) [43]. In Wuliang Mountain Black-bone chickens, association analysis between the polymorphisms of the CCAAT enhancer binding protein alpha (*C/EBPα*) gene and meat quality traits revealed that a SNP, g.552G > A, is positively correlated with IMF levels in the breast muscle. Such findings suggested that this SNP could potentially serve as a valuable molecular marker for the marker-assisted selection in chickens [44]. In Gushi-Anka F2 chickens, the lipid metabolism-related gene stanniocalcin 2 (*STC2*) was found to be highly expressed in breast muscle with high IMF content. It was suggested that *SCT2* may improve meat quality by altering the ratios of long-chain unsaturated fatty acids (LC-PUFAs) and glycerophospholipids [45]. These studies have identified key pathways and genes regulating IMF content, thereby enriching the genetic basis for individual variations across different chicken breeds.

#### IMF deposition variation among tissues

The IMF content in leg muscles has been suggested to be significantly greater than that in breast muscles. These muscles constitute the majority of chicken meat products [29]. A comparative analysis of differentially expressed genes (DEGs) between the breast and leg muscles of Beijing-you chickens revealed that IMF deposition could be influenced by cell junction-related signaling pathways, such as focal adhesion and ECM‒receptor interactions (Table 1). *PPARG* and its downstream genes, including *LPL*, *FABP4*, *THRSP*, *FABP3*, and carnitine palmitoyltransferase II (*CPT2*), were identified as playing a crucial role in these process [46]. In Wenchang chickens, a comparative gene expression analysis between breast muscle and abdominal adipose tissue revealed that pyruvate and citric acid metabolism play important roles in IMF deposition. These processes were identified as being closely associated with the expression of genes such as glyceraldehyde-3-phosphate dehydrogenase (*GAPDH*), lactate dehydrogenase A (*LDHA*), glutathione peroxidase 1 (*GPX1*), and 1,4-alpha-glucan branching enzyme 1 (*GBE1*). In contrast, abdominal fat (AF) deposition was reported to depend on fatty acid synthesis and lipid droplet formation, which are regulated by genes such as fatty acid binding protein 1 (*FABP1*), ELOVL fatty acid elongase 6 (*ELOVL6*), *SCD*, and *ADIPOQ* [47]. Another integrated analysis comparing breast muscle and abdominal adipose tissue of Lushi chickens revealed significant differences in the lipid compositions between IMF and AF. It was identified that the upregulated metabolites in IMF were mainly glycerophospholipids, such as phosphatidylcholines, phosphatidylethanolamines, phosphatidylglycerols, and phosphatidylinositol, while those in AF were primarily glycerolipids, including triacylglycerols and diacylglycerols. Subsequent in vivo and in vitro experiments confirmed that the transmembrane protein 164 (*TMEM164*) gene might be mainly involved in the positive regulation of IMF deposition and have a certain negative regulatory effect on AF deposition. Meanwhile, the zinc finger protein 488 (*ZNF488*) gene was found to have a potential unique positive regulatory function on AF deposition [48]. These studies have elucidated the histological variations and molecular mechanisms underlying fat deposition in chickens.

#### IMF deposition differences among developmental stages

Several studies have reported significant differences in IMF levels in chickens at various developmental stages [49–54]. These differences provide an ideal model for investigating the molecular mechanisms underlying IMF deposition. In Beijing-you chickens, a proteome analysis conducted at 1, 56, 98, and 140 d identified apolipoprotein A1 (APOA1) and heat shock protein family B (small) member 1 (HSPB1) as potential biomarkers for IMF deposition in breast muscle (Table 1) [49]. Integrated proteomic and metabolomic analysis revealed that protein processing and PPAR signaling pathways are involved in promoting IMF deposition. This finding was observed when comparing Beijing-you chickens and Cobb broilers at embryonic day (ED) 12, ED 17, and 1 d and 14 d post-hatch [50]. A transcriptome analysis conducted from 3 to 18 weeks in Beijing-you chickens discovered that genes related to energy metabolism, including acyl-CoA thioesterase 9 (*ACOT9*), cholesteryl ester transfer protein (*CETP*), lipin 1 (*LPIN1*), diacylglycerol o-acyltransferase 2 (*DGAT2*), retinol binding protein 7 (*RBP7*), fructose-bisphosphatase 1 (*FBP1*), and phosphorylase kinase regulatory subunit alpha 1 (*PHKA1*), are likely to play a role in regulating IMF deposition in breast muscle [51]. In thigh muscles of Beijing-you chickens, a proteomic analysis conducted at 150, 300, and 450 d revealed that the IMF transport and deposition process is accompanied by the processes of PPAR, MAPK, focal adhesion, and ECM–receptor interactions signaling pathways. Proteins such as apolipoprotein C3 (APOC3), APOA1, stathmin 1 (STMN1), HSPB1, p21 (RAC1) activated kinase 2 (PAK2), TGF-β, apoptosis-inducing enzyme caspase-3 (CASP3), COL1A2, collagen type IV alpha 1 chain (COL4A1), laminin subunit beta 4 (LAMB4), and THBS1 were found to be significantly enriched in these pathways [52]. In Jingxing-yellow chickens, a transcriptome analysis from ED 12 to 180 d identified several core genes associated with high IMF content in the breast muscle. These genes include *ENSGALG00000041996*, L3MBTL histone methyl-lysine binding protein 1 (*L3MBTL1*), TNFAIP3 interacting protein 1 (*TNIP1*), histone acetyltransferase 1 (*HAT1*), and BEN domain containing 6 (*BEND6*). Among these genes, *L3MBTL1*, *TNIP1*, *HAT1*, and *BEND6* have also been shown to be correlated with low AF content [53]. Another recent transcriptome analysis conducted at 7, 35, 63, 91, and 119 d in Jingxing-yellow chickens identified that signaling pathways, including adipocytokine, MAPK, mTOR, FoxO (forkhead box, sub-group O) and TGF-β, are associated with TG and phospholipids contents in breast muscles. Ribosomal protein S6 kinase B1 (*RPS6KB1*), BRCA1 DNA repair associated (*BRCA1*), cyclin dependent kinase 1 (*CDK1*), ribosomal protein S3 (*RPS3*), peroxisome proliferator-activated receptor gamma coactivator 1-alpha (*PPARGC1A/PGC-1α*), acyl-CoA synthetase long chain family member 1 (*ACSL1*), NADH:ubiquinone oxidoreductase subunit AB1 (*NDUFAB1*), NADH:ubiquinone oxidoreductase subunit A9 (*NDUFA9*), ATP synthase beta chain, mitochondrial (*ATP5B*), and protein kinase AMP-activated non-catalytic subunit gamma 2 (*PRKAG2*) were suggested to be candidate genes that may affect IMF deposition [55]. These studies have shown that, in general, chickens exhibit higher IMF deposition during the finisher phase compared to the rapid muscle development period (grower phase). Post-hatching, the differences in the rate of IMF deposition among different chicken breeds can be attributed to their distinct growth and developmental patterns. These findings have identified candidate genes for the enhancement and targeted regulation of chicken IMF. The developmental patterns and underlying mechanisms of IMF still require further investigation.

#### IMF deposition variations due to gender and rearing conditions

Environmental factors and gender have been suggested as potential factors that may influence IMF deposition in chickens. Several studies have reported on the impact of environmental factors and gender on IMF. In Beijing-you chickens and Lueyang black-boned chickens (Chinese indigenous breeds), caged systems have been found to be more conducive to IMF deposition than free-range systems [56, 57]. Further analysis revealed that the DEGs, particularly those genes within the PPAR signaling pathway with upregulated expression, such as angiopoietin-like 4 (*ANGPTL4*), *CD36*, *FATP1*, fatty acid transport protein 4 (*FATP4*), and perilipin 2 (*PLIN2*) in the leg muscles of caged versus free-range Lueyang black-boned chickens, are associated with IMF deposition (Table 1) [56]. Exercise volume may be one of the main reasons for the differences in IMF deposition in chickens caused by different housing systems. A recent study confirmed that free-range chickens with high levels of physical activity have lower IMF deposition than caged chickens with limited movement space [58]. In Beijing-you chickens, the interaction between gender and housing systems was found to have no impact on IMF deposition [57].

The conclusions regarding the impact of gender on IMF deposition in chickens are inconsistent. Studies in Cobb 500, Ross 308, Beijing-you chickens, Xueshan chickens, and Daheng broilers have shown that females have higher IMF contents than males [57, 59–61]. In female Daheng broilers, the upregulated expression of the *PLIN2* gene was identified as being positively correlated with IMF contents in breast muscles. Subsequent cellular experiments confirmed that *PLIN2* enhanced IMF deposition by promoting the proliferation and differentiation of intramuscular preadipocytes while inhibiting apoptosis [59]. These studies emphasized the critical roles of the PPAR signaling pathway and the *PLIN2* gene in IMF formation. Several other studies reported that gender has no impact on IMF content in chickens (e.g., White-tailed yellow native chickens and Pearl gray guinea fowl) or that it was higher in males (Ross 308) [54, 62, 63]. This may be partly due to breed-specific characteristics or the long-term reshaping of lipid metabolism pathways through natural or artificial selection. Research has shown that the interactions between breeds and gender significantly influenced the IMF levels in chickens [60]. Moreover, the impacts of the interactions between gender and environment factors on IMF deposition cannot be overlooked, even though one of the aforementioned studies showed no significant effects of this interaction on IMF in Beijing-you chickens [57]. This is a complex mechanism that requires more sophisticated models to accurately evaluate the multifaceted interactions between genetic, environmental, and physiological factors. For example, evaluating the impact of housing systems on chicken IMF content is complicated by the challenge of accounting for random errors from the consumption of exogenous substances, such as insects and grains, under free-range conditions. Developing an optimal free-range model is urgently needed to advance research on the effects of environmental factors and rearing practices on IMF deposition.

#### Cellular insights into IMF formation

With the advancement of high-throughput sequencing technology and the efforts of researchers, new breakthroughs have been made in understanding the underlying regulatory mechanism of IMF deposition in chicken at the cellular level. The first study to apply single-cell RNA sequencing (scRNA-seq) to investigate the heterogeneity of breast muscle cells in Jingxing-yellow chickens identified that genes *APOA1*, *COL1A1*, and *ADIPOQ* can serve as biomarkers for intramuscular fat cells in chickens (Table 1) [64]. A subsequent latest large-scale genetic analysis of an IMF-selected chicken population, which combined whole-genome resequencing, scRNA-seq, and transcriptome analysis, demonstrated that DNL predominantly occurs in myocytes, with only minor contributions from adipocytes, and is key to the formation of IMF in chicken muscle tissue. Fatty acid synthase (FASN) was identified as the crucial enzyme driving this process [65]. This finding reshaped the prevailing theory that DNL in poultry mainly occurs in the liver, with little to none occurring in muscle and adipose tissue [66, 67]. A causal mutation, rs315349829, located upstream of the *FASN* gene was identified. It was found to positively correlate with the key IMF components, C14:0 and C16:0 fatty acids, by upregulating *FASN* expression in chicken breast muscle tissue [65]. At cellular levels, the functions of two genes, tissue inhibitor of metalloproteinases 2 (*TIMP2*) and insulin-like growth factor binding protein 7 (*IGFBP7*), have been demonstrated. A co-culture model of chicken satellite cells and intramuscular adipocytes showed that *TIMP2* could promote IMF deposition through the ECM–receptor interaction signaling pathway [68]. *IGFBP7* was shown to promote the proliferation and differentiation of primary myoblasts and intramuscular preadipocytes in chicken. This was suggested to be achieved by the binding of transcriptional activators myoblast determination factor (MyoD), myogenin (MyoG), and C/EBPα to the *IGFBP7* promoter region [69]. These findings have enriched our insights into fat metabolism in poultry muscle tissue.

Although existing studies have elucidated the genetic basis of IMF deposition in chickens from the perspectives of interspecific and intraspecific differences, several limitations remain:

1. Current genetic mechanism studies have predominantly focused on specific genes or pathways (e.g., *FABP4* and PPARγ), yet they lack systematic integration of multidimensional regulatory networks (e.g., the interactions among SNPs, epigenetic modifications, and noncoding RNAs (ncRNAs)).
2. The explanatory power of existing molecular markers (e.g., the BB genotype of *FABP4*) for the IMF phenotype remains unevaluated, and their validation across breeds is insufficient (for example, the stability of *FABP4* expression among indigenous breeds is unclear).
3. Muscle tissue-specific mechanisms have yet to be fully elucidated, particularly whether the IMF differences between breast muscle and leg muscle stem from distinct adipogenic precursor cell differentiation trajectories or specific metabolic pathways.
4. Research on gene–environment interactions is limited, as the synergistic effects of nutritional interventions and genetic backgrounds (e.g., *PPARG* polymorphisms) remain unquantified.

Future research directions include:

1. Utilizing scRNA-seq, and multiomics technologies to elucidate the spatiotemporal dynamics of IMF deposition (e.g., the programming of MSCs during embryonic development and the temporal sequence of muscle differentiation in the growth phase).
2. Developing comparative models of muscle heterogeneity (e.g., organoids) to identify regulatory nodes of IMF heterogeneity in chickens.
3. Constructing a polygenic integration scoring model (integrating SNPs, epigenetic modifications, and ncRNA targets) and combining it with artificial intelligence models to optimize breeding strategies for “high IMF–fast growth” synergies.
4. Exploring tissue-specific gene-editing technologies (e.g., targeted clustered regularly interspaced short palindromic repeats (CRISPR) delivery systems) to provide new paradigms for the precise regulation of IMF.

By integrating multiomics technologies, elucidating the dynamic regulatory network of IMF, and analyzing its interactions with the environment, future research will further uncover the complex regulatory mechanisms of IMF and provide a solid theoretical foundation and technical support for breeding high-quality chicken meat.

### Epigenetic modifications and noncoding RNAs in IMF deposition

#### Epigenetic modifications

Epigenetic modifications of genetic material are known to include DNA cytosine methylation and N^6^-methyladenosine (m^6^A) modification of RNA. These modifications have been proven to regulate IMF deposition in chickens (Table 1). A genome-wide methylation analysis revealed that high IMF levels were highly correlated with the hypermethylation of the promoters of three genes: ATP binding cassette subfamily A member 1 (*ABCA1*), *COL6A1*, and glutathione S-transferase theta 1-like (*GSTT1L*). The expression levels of these genes were significantly negatively correlated with their methylation levels. *ABCA1* has been demonstrated to play a major role in cholesterol efflux, maintaining cholesterol homeostasis and lipid metabolism in adipocytes. Its hypermethylation and down-regulated expression may contribute to higher IMF content. *COL6A1* encodes a component of collagen, a major structural protein in muscle tissue. The hypermethylation and subsequent downregulation of *COL6A1* could affect muscle fiber properties, leading to changes in meat tenderness and texture. *GSTT1L* has been identified as a participant in glutathione metabolism, which is related to antioxidant defense and cellular aging. The hypermethylation and downregulation of *GSTT1L* may affect meat quality by influencing oxidative stability and aging processes in muscle tissue [70]. Another integrative multiomics analysis in Jingxing-yellow chickens identified phospholipase A2 group IVF (*PLA2G4F*) as a differentially methylated gene (DMG) that could be a key candidate influencing IMF deposition during chicken development. This DMG was significantly enriched in the glycerophospholipid metabolism pathway. Its downregulation has been shown to suppress the degradation of unsaturated fatty acids through multiple mechanisms, including inhibiting the activity of key enzymes involved in fatty acid catabolism (e.g., acetyl-CoA dehydrogenase, lipoxygenase, and fatty acid dioxygenase) and activating the PPARγ signaling pathway and its downstream target genes [71]. These findings reveal potential genes and pathways related to muscle development and meat quality through DNA methylation. These genes may serve as epigenetic markers and regulatory targets for evaluating meat quality in chickens.

Beyond transcriptional silencing via DNA methylation, post-transcriptional RNA modifications are emerging as an equally pivotal layer of epigenetic regulation. In Jingyuan chickens, studies on breast muscle tissues at different developmental stages have revealed that the m^6^A-induced ferroptosis pathway is a novel target for regulating IMF deposition. Leiomodin 2 (*LMOD2*) and several other m^6^A-regulated DMGs have been demonstrated to be potential regulatory factors [72]. Weighted gene co-expression network analysis and in vitro cell modeling further identified m^6^A methylation-mediated cubilin (*CUBN*), multiple EGF-like domain 10 (*MEGF10*), block of proliferation 1 (*BOP1*), and bone morphogenetic protein receptor 2 (*BMPR2*) could serve as potential candidate genes for regulating muscle development and IMF deposition [73]. A cross-tissue comparative analysis of m^6^A modifications between the breast and leg muscles revealed that enoyl-CoA hydratase 1 (*ECH1*), branched chain amino acid transaminase 1 (*BCAT1*), and cytochrome P450 family 1 subfamily B member 1 (*CYP1B1*) are key DMGs involved in the regulation of muscle lipid metabolism [74]. These studies reveal the epigenetic basis of chicken IMF regulation and establish a framework for optimizing chicken meat quality through precision breeding.

#### Noncoding RNAs

With advancements in epigenetics, recent studies have confirmed that endogenous noncoding RNAs (ncRNAs), including microRNAs (miRNAs), long noncoding RNAs (lncRNAs), and circular RNAs (circRNAs), play multiple regulatory roles in the formation and deposition of IMF in chickens [75, 76].

##### MiRNAs

The first dynamic expression profile of miRNAs from the breast muscle tissue of Gushi chickens revealed the core miRNAs in miRNA‒mRNA interaction networks, such as gga-miR-15a, gga-miR-103-3p, and gga-miR-138-2-3p. These miRNAs were found to be crucial in the regulation of chicken IMF deposition [77]. The molecular mechanisms underlying the miRNA-mediated regulation of IMF deposition, particularly during the proliferation and differentiation of intramuscular preadipocytes, have been elucidated by cellular-level studies (Fig. 2). For example, retinoid X receptor γ (*RXRG*) is targeted by gga-miR-140-5p, whereas sterol carrier protein 2 (*SCP2*), acetyl-CoA acyltransferase 1 (*ACAA1*), and acyl-CoA oxidase 1 (*ACOX1*) are targeted by miR-15a to promote the differentiation of intramuscular preadipocytes, thereby increasing IMF deposition in muscle [78, 79]. MiR-223 and gga-miR-18b-3p have been shown to target mitochondrial glycerol‑3‑phosphate acyltransferase (*GPAM*) and acyl-CoA thioesterase 13 (*ACOT13*), respectively, which inhibits this differentiation process [80, 81]. MiR-24-3p and miR-128-3p have been determined to exhibit dual regulatory functions: promoting the proliferation of intramuscular preadipocytes and inhibiting their differentiation. MiR-24-3p was reported to achieve this by blocking the expression of annexin A6 (*ANXA6*) [82], and miR-128-3p was shown to inhibit their differentiation by downregulating farnesyl diphosphate synthase (*FDPS*) expression, although the specific target genes that promote proliferation remain unidentified [83]. Gga-let-7a-3p has been reported to inhibit the proliferation and differentiation of intramuscular preadipocytes. Cellular-level transcriptomic analysis indicated that it may exert these effects through pathways such as the PPAR signaling pathway, oxidative phosphorylation, and ribosome-related processes [84]. Its specific target genes remain to be further studied. A comparative study in Beijing-you chickens at different developmental stages revealed key miRNA–mRNA pairs, such as miR-6701-3p-PTEN, miR-1563-WWP1, miR-6701-3p-BMPR1B, miR-29c-3p-PIK3R1, and miR-449c/d-5p-TRAF6, which were found to play core roles in regulating IMF deposition [85].

Oxidative myofibers (types I and IIa) or slow-twitch fibers (type I) are typically characterized by a relatively high fat content, which positively affects the IMF content [12, 86–89]. The regulatory role of ncRNAs in myofibers has also emerged as a key research focus, as depicted in Fig. 3. In a study on the mRNA–miRNA transcriptomes of Qingyuan partridge chickens (an indigenous Chinese breed), miR-499-5p and miR-196-5p were identified as the two most abundant and upregulated miRNAs in the oxidative muscle sartorius, compared to the glycolytic muscle (pectoralis major). Further experiments confirmed that miR-499-5p targets *SOX6*, a specific factor known to inhibit the expression of slow-twitch muscle genes, and miR-196-5p targets *CALM1*, a key element in the cGMP-PKG and calcium signaling pathways. These interactions collectively contribute to the regulation of slow-twitch fiber formation in muscles [90]. It has been found that *PPARGC1A* is a transcriptional coactivator that drives the transformation of fast- to slow-twitch fibers via various biological pathways (e.g., glycolysis, the TCA cycle, and fatty acid β-oxidation). Experiments at both the individual and cellular levels showed that miR-193b-3p could inhibit the expression of *PPARGC1A* by directly binding to its 3′ UTR, thereby inducing the fast-twitch muscle phenotype [91]. In another study, miR-1611 was demonstrated to be upregulated in leg muscles that are rich in slow-twitch fibers and could target the SIX homeobox 1 (*Six1*) gene, facilitating the transition from fast- to slow-twitch fibers. MiR-1611 was determined to be a direct target within the ceRNA network involving lncRNA*-Six1* and *Six1*. LncRNA*-Six1* regulated the expression of *Six1* and the transition of fiber types by competing with miR-1611 for binding sites [92]. These findings highlight the key mechanisms underlying the miRNA-mediated regulation of chicken IMF deposition and reveal their potential for targeted regulation of meat quality.

##### LncRNAs and circRNAs

LncRNAs constitute a novel class of regulatory RNA molecules that are transcribed by RNA polymerase II and exceed 200 nucleotides in length [92, 93]. They have been reported to perform various biological functions primarily by acting as competing endogenous RNAs (ceRNAs), regulating gene expression, and encoding small peptides (Figs. 2 and 3). In Gushi chickens, lncRNA *IMFNCR* has been shown to act as a molecular sponge for miR-128-3p and miR-27b-3p through the ceRNA mechanism, thereby alleviating the suppression on the expression of *PPARG* by these miRNAs and facilitating the differentiation of intramuscular preadipocytes [94]. Skeletal muscle development has been found to be promoted by miR-128-3p and miR-27b-3p through targeting the myostatin (*MSTN*) gene, which facilitates the differentiation of satellite cell and the proliferation of myoblast [94–96]. The lncRNA *IMFNCR* might be one of the ‘switches’ regulating chicken muscle development and IMF deposition. With respect to *cis*-regulatory mechanisms, *lncAD* in Gushi chickens has been shown to be coexpressed with multiple genes in the PPAR signaling pathway and to promote the differentiation of intramuscular preadipocytes via *cis*-activating thioredoxin reductase 1 (*TXNRD1*) [97]. The differentially expressed lncRNA-46546 between Rose Crown and Cobb broiler embryos has been discovered to promote TG synthesis and lipid droplet accumulation in intramuscular preadipocytes through cis-regulating its target gene, 1-acylglycerol-3-phosphate-O-acyltransferase 2 (*AGPAT2*) [98]. In Lushi chickens, *LncHLFF* has been identified as a molecular sponge for miR-2188-3p through the ceRNA mechanism, promoting the post-transcriptional expression of GATA binding protein 6 (*GATA6*) and encoding functional micropeptides to enhance hepatic lipid synthesis. Additionally, it has been shown to facilitate ectopic fat deposition in muscles via hepatocyte-adipocyte communication mediated by exosomes without altering AF deposition [99]. To our knowledge, this is the first study in which lncRNAs were reported to promote ectopic IMF deposition in chickens.

LncRNAs has been detected that can regulate myofiber types through interactions with proteins (Fig. 3). For instance, myosin heavy chain 1B (MYH1B) was confirmed to be bound by lncRNA*-FKBP1C*, which led to an increase in its protein stability, inhibited myoblast proliferation and apoptosis, and promoted their differentiation and transition to slow-twitch fibers [100]. The protein stability and activity of ATPase sarcoplasmic/endoplasmic reticulum Ca^2+^ transporting 2 (SERCA2) were enhanced by lnc*-EDCH1* through interaction, which helps maintain Ca^2+^ homeostasis and activates the protein kinase AMP-activated catalytic subunit alpha 1 (AMPK) pathway, thereby improving mitochondrial efficiency and activating the slow-twitch myofiber phenotype [81]. The activation of acetyl-CoA carboxylase alpha (ACACA) was induced and the activity of pyruvate carboxylase (PC) was inhibited by the lncRNA *ZFP36L2-AS*. These actions repressed the oxidation of fatty acids, facilitated the deposition of IMF, and activated the generation of a fast-twitch muscle phenotype [101]. A switch from slow- to fast-twitch fibers was driven by the lncRNA *MYH1G-AS* through the inhibition of fibroblast growth factor 18 (FGF18) protein stabilization. This reduction in FGF18 protein stabilization decreases its interaction with SNF2 related chromatin remodeling ATPase 5 (SMARCA5), thereby repressing the chromatin accessibility of the SMAD family member 4 (*SMAD4*) promoter and activating the SMAD4-dependent pathway [102]. Exploring the nutritional substances that interact with these lncRNAs would provide new strategies for the targeted regulation of muscle development and IMF deposition.

CircRNAs are also a class of ncRNAs that are widely present in various tissues and cells [103]. Its roles have been confirmed in multiple physiological processes in chickens, including follicular development [104, 105], bursal development [106], AF deposition [107], and skeletal muscle development [108]. Specific circRNAs, such as circARMH1, circLCLAT1, circFNDC3AL, and circCLEC19A, have been identified through the integrated analysis of RNA and miRNA sequencing data from preadipocytes of muscle and adipose tissue in chickens (Fig. 2). These circRNAs potentially influenced adipogenesis by regulating miRNAs via PPAR and fatty acid metabolism-related pathways [109]. In Gushi chickens, *circITGB5* has been confirmed to promote the expression of the downstream target gene *CPT1A* by adsorbing miR-181b-5p, thereby suppressing the proliferation and differentiation of intramuscular preadipocytes [110]. Current research on the regulation of chicken IMF deposition by circRNAs remains limited, and further studies are warranted to elucidate its regulatory mechanism.

The regulatory role of circRNAs on myofiber types has also been confirmed (Fig. 3). The differentially expressed *circPTPN4* between the pectoralis major and soleus muscles of Xinghua chickens has been shown to act as a ceRNA, regulating the expression of nicotinamide phosphoribosyltransferase (*NAMPT*) by adsorbing miR-499-3p. This interaction activated the AMPK signaling pathway, thereby promoting the proliferation and differentiation of myoblasts and activating the fast-twitch fiber phenotype [111]. These results indicate that ncRNAs, which govern the transition of chicken myofiber types through diverse mechanisms, may also serve as potential targets for modulating IMF and enhancing meat quality in chickens.

## Nutritional regulation on IMF deposition and meat quality

Appropriate nutritional interventions could improve the meat quality and body composition of broiler chickens. These interventions involve nutrients that encompass both the fundamental constituents of the product and the bioactive compounds that modulate gene expression, signaling pathways, metabolic processes, and physiological functions. In oviparous chickens, post-hatching nutrients, which include energy, protein, amino acids (AAs), vitamins, fatty acids, and exogenous plant extracts, are derived primarily from the diet. Pre-hatching nutrition, or embryonic nutrition, includes maternally derived nutrients as well as those provided through IOF.

### Dietary energy, protein and amino acids

#### Energy

The metabolizable energy (ME) content of the diet has been shown to significantly influence the body composition of chickens [6]. An increase in the dietary ME level was shown to be correlated with improvements in IMF deposition and meat quality. For instance, in Huxu yellow-feathered chickens, an indigenous Chinese breed, the diameter and cross-sectional area of breast myofibers were observed to decrease linearly, and the myofiber density was increased with increasing ME intake during the finisher phase. These changes enhanced the IMF content and tenderness of the chicken meat. The optimal quality and flavor of chicken meat were achieved when the dietary ME level reached 3,180 kcal/kg. This increase is likely linked to the consumption of a high-energy diet, which appears to promote the mRNA expression of the *FABP3* and *APOB* genes, thereby facilitating the increased IMF deposition in muscle tissues [112]. However, diets with increased ME might also decrease feed utilization efficiency, as an increased AF rate has been exhibited [113]. This contradiction can be explained by the dual effects of high-energy diets. While such diets have been shown to promote IMF deposition, they also led to excessive fat accumulation in non-target areas, such as the abdomen. Excessive AF is known to be associated with a reduction in the economic value of the carcass, as it does not contribute to commercial value. High-energy diets were also suggested to increase the metabolic burden on chickens, potentially altering energy allocation. This shift may result in more energy being used for fat synthesis and storage rather than for growth and production, which could contribute to a decrease in feed conversion efficiency [114]. These findings suggest that relying solely on high-ME diets to improve meat quality may not be the most effective strategy. Future research should optimize feed formulations to enhance meat quality while minimizing the adverse effects on feed utilization efficiency.

Research findings on the impact of low-ME diets on chicken IMF deposition and meat quality are inconsistent. For example, in Ross 308 broilers, diets with ME levels 23.9 kcal/kg and 59.75 kcal/kg below the control diet were found to significantly increase the IMF content, pH_15min_, meat color L^*^ values and antioxidant capacity, but also to negatively affect the WHC of the breast muscle [115]. A reduction in dietary ME by 100 kcal/kg relative to the control group was reported to not influence meat quality parameters, including WHC, pH, meat color L^*^ values, and tenderness [116, 117]. Up to now, research on the effects of low-energy diets on IMF deposition and meat quality in chickens remains limited. Future research should investigate the interaction between dietary energy levels and various factors such as genetic background, age, and breed, and assess how these interactions might influence IMF deposition and meat quality.

#### Crude protein

The conclusions are inconsistent across several studies on the effects of dietary crude protein levels in terms of meat quality (Table 2). IMF and meat quality in chickens have been shown to be influenced by low-protein diets. In a comparative study, it was found that an isoenergetic diet containing 17% CP was associated with improvements in WHC, pH_24h_, and meat color a^*^ and L^*^ values in the breast muscle of lean-line chickens, when compared to a diet containing 23% CP [7]. Reducing the CP content of diets to 18% for the starter phase and 16.5% for the finisher phase was found to decrease the IMF content in broiler muscle, which in turn might have affected the palatability of chicken meat [118]. In Ross 308 broilers, a study reported that the quality indicators of breast muscle were not altered by a reduction in dietary CP levels from 23% to 21% for starters and from 20% to 18% for finishers under isoenergetic conditions [119]. Another study showed that an increase in breast meat yield and an improvement in meat quality were observed when the diet CP was reduced by 1.5% while maintaining a constant nitrogen-corrected apparent metabolizable energy (AMEn) [120]. Lower CP diets (18.0% vs. 19.1% for starter; 16.5% vs. 17.7% for finisher) were shown to have an adverse effect on quality indicators, such as cholesterol content, WHC, and meat color, regardless of the breed—fast-growing (Ross 308), medium-growing (Hubbard JA757), or slow-growing (ISA Dual) [121].

**Table 2:** Effects of dietary protein and amino acids on broiler meat quality

Dietary protein and amino acid levels^a^		Chicken breeds	Meat quality^e^	References
Starter	Finisher			
**CP**				
21.7%	17%	Lean-line	WHC ↑, pH_24h_ ↑, meat color a^*^ and L^*^ values ↑	[7]
18%	16.5%	Ross PM3	IMF ↓	[118]
21%	18%	Ross 308	Unchanged meat quality	[119]
21.33%	17.41%	Ross 308	Breast meat yield ↑, drip loss ↓	[120]
18.0%	16.5%	Ross 308, Hubbard JA757, ISA Dual	cholesterol content ↑, WHC ↓, meat color ↓	[121]
15% CP with 11.51 MJ/kg ME		Beijing-you	IMF and flavor ↑	[6]
15% CP with 12.56 MJ/kg ME		Taihe Silky Fowl	IMF ↑, tenderness ↑, flavor ↑, and FCR ↓	[122]
21.39%	16.5%^b^	Ross 308	Cooking lose ↓, meat color b^*^ values ↓	[124]
23.1%	16%^b^	Ross PM3	pH ↑, lightness ↓, drip loss ↓	[123]
**Met**				
0.65%	0.57%	Arbor Acres, Partridge Shank	pH ↑, meat color L^*^ value ↓, cooking loss ↓	[8]
0.04%, 0.12%, 0.32% DL-Met or Met dipeptide	0.04%, 0.12%, 0.32% DL-Met or Met dipeptide	Ross 308	pH ↑, meat color L^*^ value ↓, cooking loss ↓; dose-dependent	[125]
0.28% DL-Met or 0.29% DL-MM	0.27% DL-Met or 0.28% DL-MM	Cobb 500	IMF ↓, meat color a^*^ value ↓, antioxidant capacity ↑, lipid peroxidation ↓	[126]
**Lys**				
1.15%	0.85%	Erlang Mountainous	Carcass weight ↑, muscle weight ↑ Unchanged meat quality	[132]
Lys and Met exceeded NRC (1994) recommendation by 40%		Ross 308	IMF ↓, abdominal fat ↓	[133]
14 g/kg Lys	7.7 g/kg Lys combined with AA −^c^	Ross PM3	pH ↑, dark meat↑, WHC ↑	[134]
14 g/kg Lys	7.7 g/kg Lys combined with AA +^c^		pH ↓, light meat ↑, WHC ↓	
—	15% CP combination with 0.19% Met and 0.25% Lys	Hubbard	Unchanged carcass yield and meat composition	[135]
**Arg**				
100.39% Arg of the Ross 308 Nutrition Specifications^d^		Ross 308	Muscle yield ↑, unchanged abdominal fat	[136]
168% dArg of the Ross catalog		Ross broilers	Muscle weight and IMF ↑, abdominal fat ↓ Plasma TC and TG ↓	[137]
**Trp**				
0.27% Trp during the last 3 weeks before marketing		Abore Acres	Drip loss ↓	[139]
**BCAA**				
20% CP, 2 g/kg L-Leu or 0.1g/kg HMB	19.98% CP, 1.65% L-Leu	Ross 308	Muscle yield ↑, WHC ↑, pH ↑ Meat color ↑	[148]
150% Ile and Val of the NRC (1994) requirements		Cobb	Free glutamine ↑	[149]
22% or 20% CP, Leu:Ile:Val ratio of 4:1:1	20% CP	Ross 308	Breast meat yield ↓	[150]

^a^The values represent the content or level of addition in the diets during the starter or finisher phases, unless otherwise specified. *Arg* Arginine, *BCAA* Branched chain amino acids, *CP* Crude protein, *DL-Met* DL-methionine, *DL-MM* DL-methionyl-DL-methionine, *dArg* Digestible arginine, *FCR* Feed conversion ratio, *HMB* β-hydroxy-β-methylbutyrate, *Ile* Isoleucine, *L-Leu* L-leucine, *Lys* Lysine, *ME* Metabolizable energy, *Met* Methionine, *NRC* National Research Council Nutrient Requirements of Poultry, *Trp* Tryptophan, *Val* Valine; —: Not mentioned

^b^Dietary amino acids were supplemented to compensate for the reduced amino acid intake caused by low-protein diets

^c^AA − or AA + : A low or high amount of amino acid (AA) relative to other AA, calculated in relation to Lys and corresponding respectively to 90% or 110% of the ideal AA profile proposed by Mack et al. [297] for nutritionally essential AA in finishing broiler chickens

^d^This value was derived from the established quadratic regression models

^e^*IMF* Intramuscular fat, *pH*_*24h*_ The pH of meat is measured 24 h after slaughter, *TC* Total cholesterol, *TG* Triglycerides, *WHC* Water-holding capacity; ↑: Increased or improved compared with the control group without treatment; ↓: Decreased or diminished compared to the control group without treatment

Recent research has confirmed that the ratio of dietary energy to protein can significantly affect muscle protein and fat levels (Table 2). For example, in Beijing-you chickens, a diet containing 11.51 MJ/kg of ME and 15% CP was shown to maximize the improvement of IMF levels and flavor in the breast meat [6]. In Taihe silky fowl (an indigenous Chinese breed), a diet with 12.56 MJ/kg of ME and 15% CP was found to optimize the content of IMF, tenderness, flavor, and feed conversion ratio (FCR) [122]. These varying results are likely attributed to interactions between energy and protein intake, as well as the supplementation of essential amino acids and the balance among amino acids. Amino acids are fundamental not only for muscle protein synthesis but also as essential regulatory factors in metabolic pathways, antioxidant systems, and enzymatic processes [8, 9]. Optimizing the dietary amino acid profile is crucial for mitigating the negative effects of low-protein diets. Studies have shown that the impairment of production performance, muscle composition, and meat quality characteristics in broiler chickens could be prevented by achieving amino acid balance, for instance by adjusting the ratio of digestible threonine (dThr) to digestible lysine (dLys) and the ratio of digestible arginine (dArg) to dLys, even when the dietary CP content was reduced to 17% or lower. In fact, such adjustments have been found to improve meat quality [123, 124]. To ensure meat quality under low-protein diets, it is deemed essential not only to balance the dietary energy-to-protein ratio but also to meet the requirements of essential amino acids, such as methionine (Met), Thr, and Arg, as well as nonessential amino acids, such as glycine (Gly) [123].

#### Methionine

Met is considered the first limiting amino acid in corn-soybean meal diets for chickens and has been suggested to significantly impact the meat quality of broilers (Table 2). Studies have found that increasing the dietary Met content to 0.65% during the starter phase and to 0.57% during the finisher phase significantly increased the pH, decreased the meat color L^*^ value, and reduced the cooking loss of breast muscle [8]. This effect appeared to be almost dose dependent and was not influenced by the source of Met, whether it was DL-Met or a Met dipeptide [125]. When dietary Met was supplemented, reductions in IMF deposition and the a^*^ value of meat color were observed, along with enhancements in the antioxidant capacity of breast muscle and alleviation of lipid peroxidation caused by heat stress [8, 126]. Met supplementation has been demonstrated to have positive effects on chicken meat quality to some extent, although it might reduce IMF deposition. It has been proposed that Met might reduce the activity of pyruvate kinase, which could inhibit glycolysis and lactate accumulation. Such an action might increase the pH of the breast muscle. The L^*^ value and cooking loss of the meat were found to be decreased [127–129]. The increased antioxidant capacity of the breast muscle may be attributed to its contribution to the biosynthesis of the endogenous antioxidant glutathione [8, 130].

#### Lysine, arginine and tryptophan

Lys is recognized as the second limiting amino acid after Met in the basal diet for chickens [131]. As displayed in Table 2, supplementing diets with Lys alone has been shown to significantly improve broiler production performance, such as increasing carcass weight, breast muscle weight, and leg muscle weight, without altering meat quality indicators such as meat color, pH, and the IMF content [132]. The interactions between Lys and other amino acids have been observed to have significant impacts on meat quality. A linear reduction in body fat deposition was observed in Ross 308 broilers when diets exceeded the National Research Council (NRC 1994) recommendations for Lys and Met. Particularly, when Lys and Met levels were increased by 40% above the recommendations, fat in the breast and leg muscles was reduced by 35% and 27%, respectively, along with a corresponding decrease in AF [133]. Meat quality characteristics have also been observed to be influenced by the degree of amino acid balance in the diet. For instance, when the levels of other essential amino acids in the diet were reduced (AAs −), meat with a relatively high pH (pH > 6.0), darker color, and greater WHC could be resulted from a lower Lys level. Conversely, meat with a lower pH (pH < 5.85), lighter color, and poorer WHC could be resulted from a combination of a lower Lys level and increased levels of other essential amino acids (AAs +) [134]. Under a low-protein diet, the carcass yield and percentages of crude protein, total fat, and moisture in the breast muscle of broilers have been shown to be maintained with adequate supplementation of Lys and Met, even when the dietary crude protein level was 15% [135]. These findings underscore the regulatory effects of the interaction between Lys and other amino acids on meat quality traits, as well as the importance of amino acid balance in broiler nutrition and management.

Appropriate Arg supplementation has been shown to have positive effects on muscle development and IMF deposition in broiler chickens (Table 2). A dietary Arg level of 100.39% of the recommended amount in the Ross 308 Nutrition Specifications was indicated to maximize muscle yield without affecting AF deposition [136]. Lipid metabolism patterns in broilers were detected to be altered when the intake of Arg exceeded the recommended levels. For instance, increasing the dietary dArg concentration to 168% of the recommended level was shown to not only increase the weight and IMF content of the breast muscle but also reduce AF deposition and plasma levels of cholesterol and TG in broilers. These results could be attributed to the fact that Arg supplementation upregulated the expression of genes associated with fat synthesis in muscles, such as *FASN* and *LPL*, whereas these genes were down-regulated in AF. This dual action modulated the distribution of fat across body tissues [137, 138].

Feeding Arbor Acres broilers a diet with a high tryptophan (Trp) level (Trp, 0.27%) during the last 3 weeks before marketing has been reported to tend to reduce the drip loss of breast meat. It was suggested that a high level of Trp might enhance the juiciness of chicken meat (Table 2) [139]. This promoting effect could be attribute to Trp's ability to maintain the functional integrity of muscle cell membranes and activate antioxidant enzymes such as glutathione peroxidase and catalase. These actions reduced the loss of intracellular water and inhibit lipid peroxidation and muscle cell membrane damage, thereby potentially improving meat quality [139–142].

#### Branched-chain amino acids (*BCAAs*)

BCAAs, including leucine (Leu), isoleucine (Ile), and valine (Val), are acknowledged as constituting an important class of essential amino acids in muscle tissue [143–145]. The transamination of BCAAs primarily occurs in skeletal muscle. Research has indicated that the mTOR signaling pathway in the skeletal muscles of neonatal chicks could be activated by a high Leu diet [146], and this activation promoted protein synthesis while inhibiting its degradation [147]. Similar findings have also been consistently confirmed in both animal and human studies [13]. The regulation of chicken meat quality has been shown to be influenced by Leu and its derivatives (Table 2). In Ross 308 broilers, it was shown that the adverse effects of low-protein diets on growth, development, and meat quality, including muscle yield, WHC, pH, and meat color (a^*^, b^*^, and L^*^), were reversed when the diets were supplemented with Leu or its metabolite β-hydroxy-β-methylbutyrate [148]. Adjusting the ratio of BCAAs in the diet, especially increasing the dietary levels of Ile and Val to 150% of the required amount was suggested to increase the concentration of free glutamine, a primary flavor component, in the breast meat of broilers [149]. Under heat stress conditions, the yield of broiler breast meat was found to decrease when additional dietary BCAAs (1.0, 0.25, and 0.25 g/kg of L-Leu, L-Ile, and L-Val, respectively) were supplemented. This negative impact might be due to the antagonistic effects between Ile and Val [150]. Although antagonistic effects among BCAAs have been noted in broilers, the underlying mechanisms remain to be fully elucidated [151, 152]. Future research is needed to investigate the interactive mechanisms among BCAAs and their impact on meat quality to inform more targeted strategies for enhancing meat quality in broilers.

### Fatty acids

#### Ratios of omega-6 to omega-3 polyunsaturated fatty acids (PUFAs)

Omega-6 (ω-6) and omega-3 (ω-3) PUFAs are essential LC-PUFAs. In modern diets, a deficiency of ω-3 PUFAs is leading to an imbalance in the intake ratio of ω-6/ω-3 PUFAs, which is considered to pose a challenge for human health. The need for producing poultry meat rich in ω-3 PUFAs has gained attention among researchers [153]. It has been determined that the content of ω-3 PUFA, including alpha-linolenic acid (ALA), eicosapentaenoic acid (EPA), and docosahexaenoic acid (DHA) in muscle tissue could be significantly increased, and the ω-6/ω-3 PUFAs ratios could also be reduced, with the dietary supplementation of ω-3 PUFA sources [10]. These desired effects could be achieved by supplementing with 1% fish oil, canola oil, or flaxseed oil [154], 5.5% canola oil [155], or 1%–4% DHA-rich microalgal biomass [156].

Some adverse effects on IMF deposition and meat quality have been observed in several studies, as shown in Table 3. For example, the addition of 2.5% fish oil was observed to significantly increase the drip loss and thawing loss of muscle in Arbor Acres broilers [157]. The incorporation of 0–1% flaxseed oil was noted to decrease the tenderness of breast muscle in Beijing-you chickens [158]. The inclusion of 2% DHA-rich microalgae was seen to significantly reduce the IMF content of breast muscle in Cornish broilers [159]. Similar effects have also been documented with other sources of ω-3 PUFA [160–162]. These findings suggest that balancing the effects of ω-3 PUFAs on IMF and meat quality is necessary when using these compounds as supplements. The reduction in the IMF content might be partially attributed to ω-3 PUFAs altering the lipid metabolism pattern in chickens. Because the supplementation of ω-3 PUFAs has been detected to reduce the AF deposition in broilers [157, 160, 161, 163], and to downregulate the expression of key regulators in the de novo fatty acid synthesis pathway (such as *ACACA*, *FASN*, and *SCD*) and β-oxidation pathway (such as *CPT2* and *ACS*), as well as the regulators involved in fatty acid metabolism, such as sterol regulatory element binding protein 1 (*SREBP1*), fatty acid desaturase 1 (*FADS1*), *FADS2*, *ELOVL2*, and *ELOVL5* in the liver [156].

**Table 3:** Effects of dietary supplementation with omega-3 PUFAs on broiler meat quality^a^

Supplementation levels and sources^b^	ω-6/ω-3 ratios		Chicken breeds	Effects^c^	References
	**Starter**	**Finisher**			
2.5% fish oil	6.82	5.62	Arbor Acres	Drip loss and thawing loss↑	[157]
0.12% flaxseed oil 0.42% flaxseed oil 1.00% flaxseed oil	16.28 8.57 4.46	19.31 10.74 5.51	Beijing-you	Tenderness of breast muscle ↓	[158]
2% DHA-rich microalgae	10.77	10.51	Cornish	IMF ↓	[159]
20 g/kg schizochytrium powder 20 g/kg salmon oil 20 g/kg flaxseed oil	5.54 5.26 1.99	7.72 6.72 1.98	Ross broilers	Antioxidant capacity of muscle on the 7 th d of storage ↓	[164]
74 g/kg DHA Gold™	—	2:1–1:1	Ross 308	Muscle oxidation within 48 h post-mortem ↑	[165]
1 g/kg *Chlorella vulgaris* 1 g/kg *Spirulina platensis* 1 g/kg *Amphora coffeaeformis*	—	—	Cobb 500	MDA and protein carbonyl ↓, SOD activity ↑	[167]
2%−2.25% fish oil 2%−2.25% flaxseed oil	4:1 2.5:1	4:1 2.5:1	Ross 308	Final body weight ↑, FCR ↑, PER ↑, dressing percentage ↑, muscle percentage ↑	[160]
4% DHA-rich *Aurantiochytrium acetophilum* biomass (6.8 g DHA/kg diet)	2.78:1	2.20:1	Cornish	BWG ↓, FCR ↓, and breast muscle weight ↓	[168]

^a^*PUFAs* Polyunsaturated fatty acids

^b^*DHA* Docosahexaenoic acid; DHA Gold™ is a product derived from Schizochytrium marine algae with a golden hue

^c^*BWG* Body weight gain, *FCR* Feed conversion ratio, *IMF* Intramuscular fat, *MDA* Malondialdehyde, *PER* Protein efficiency ratio, *SOD* Superoxide dismutase; ↑: Increased or improved compared with the control group without treatment; ↓: Decreased or diminished compared with the control group without treatment

A lower dietary ω-6/ω-3 PUFA ratio may be detrimental to the antioxidant capacity of muscle tissue (Table 3). Studies have shown that the antioxidant capacity of muscle on the 7^th^ d of storage was significantly reduced when the ratio was adjusted to 6:1–2:1 [164]. Further reducing the ratio to 2:1–1:1 has been found to accelerate muscle oxidation within 48 h post-mortem [165]. This phenomenon might be attributed to the oxidation susceptibility of ω-3 PUFAs and their accumulation in muscle tissue. When supplementing with ω-3 PUFAs, it is important to stabilize through combination with antioxidants like vitamin E or soybean isoflavones to improve meat product stabilities [163, 166]. An appropriate supplementation of ω-3 PUFAs seems to be beneficial to the antioxidant performance of chicken meat. For example, diets supplemented with 1 g/kg of microalgae, such as *Chlorella vulgaris*, *Spirulina platensis*, or *Amphora coffeaeformis*, have been discovered to reduce the malondialdehyde (MDA) and protein carbonyl levels in breast muscle and enhance superoxide dismutase (SOD) activity [167].

The source of dietary ω-3 PUFAs has also been detected to significantly impact the growth performance of broilers (Table 3). Studies have shown that the improvements in the production performance of broilers, including their final body weight, FCR, protein efficiency ratio, dressing percentage, and muscle percentage, were achieved when using fish oil or flaxseed oil as a source of ω-3 PUFAs and the dietary ω-6/ω-3 PUFAs ratio was adjusted to 4:1 or 2.5:1 [160]. It was observed that using DHA-rich *Aurantiochytrium acetophilum* biomass as an ω-3 PUFA source and adjusting the ratio to approximately 3:1 could negatively affect body weight gain (BWG), FCR, and breast muscle weight, and downregulate the expression of key signaling molecules in muscle protein synthesis, including the mRNA levels of *mTOR* and ribosomal protein S6 (*S6*), as well as the protein ratios of p-S6/S6 and p-S6 Kβ1/S6 Kβ1 [168]. A fishy odor in chicken meat was supposed to be induced when fish-derived products were used as PUFA sources, which might reduce market acceptance [165, 169, 170]. When selecting ω-3 PUFA supplements, it is necessary to consider the impact of their source on the growth performance and product acceptability.

In practice, the source, type, and dosage of ω-3 PUFAs, as well as their interactions with other nutrients in the diet should be taken into account to optimize broiler growth performance and product characteristics. Adjusting dietary ω-6/ω-3 PUFA ratios or combining them with specific amino acids has been suggested to further improve IMF and meat quality [158, 159]. The authors conclude that a dietary ω-6/ω-3 PUFA ratios between 4:1 and 2.5:1 is ideal, but attention should be given to the source of ω-3 PUFAs and the supplementation of antioxidants. Considering the promoting effects of LC-PUFAs on the proliferation of muscle stem cells (MuSCs) [171] and the differentiation of FAPs [172], future research should explore the regulatory mechanisms of LC-PUFAs on muscle development and adipogenesis in chickens at the cellular level.

#### Conjugated linoleic acids

Conjugated linoleic acids (CLAs) are positional isomers of linoleic acid [173], and have been confirmed to perform multiple biological functions, such as regulating lipid metabolism and preventing cancer, atherosclerosis, diabetes, and obesity in the body [174–178]. In chickens, diets supplemented with 1.5%–2% CLAs have been found to significantly alter the fatty acid composition in muscle tissue. It was detected that these diets increased the content of CLAs and saturated fatty acids [179–182] and the ratio of ω-6/ω-3 [183], along with a decrease in the levels of monounsaturated and polyunsaturated fatty acids [182, 184]. The effects of CLAs on chicken IMF content have been observed to dose-dependent. Low concentrations (0.25%–1%) of CLAs were observed to promote IMF deposition [185, 186], whereas high concentrations (1.8%–2%) were found to have the opposite effects [179, 185]. An appropriate supplementation of CLAs has been shown to have positive effects on the improvement of chicken meat quality. It was shown that diets supplemented with 0.25%–2% CLAs reduced the shear force and b^*^ value of breast muscle [185, 186], and that 1.5% CLAs enhanced the antioxidant capacity of chicken meat [181]. Potential interactions have been detected between CLAs and dietary lipids. Compared to supplementation with 4.2% CLAs alone, a diet supplemented with 2.1% CLAs and 3.5% soybean oil was found to be more effective in promoting the deposition of CLAs in chicken muscle in a previous study [187]. Further research has also confirmed that the increased deposition of CLAs in muscle was not merely the result of lipid accumulation [188]. It has been shown that the reduction in the content of polyunsaturated fatty acids and the increased ω-6/ω-3 ratio induced by CLAs in muscle could be alleviated by incorporating CLAs and ω-3 fatty acids into the diet [189, 190]. During the starter phase, the BWG of broilers was shown to be negatively affected by dietary supplementation with 1% CLAs, and increasing the dietary energy level was observed to be ineffective in compensating for this impact [191]. An accurate level of CLAs is considered essential for optimal broiler production. Future studies should optimize CLAs supplementation and investigate its interactions with other nutrients to enhance fatty acid utilization and to improve the balance of fatty acids in chicken meat.

### Phytochemicals

Phytochemicals are natural chemicals extracted from plants that are rich in a variety of bioactive components, including micromolecules such as polyphenols, flavonoids, alkaloids, as well as macromolecules like saponins [192]. These compounds have been confirmed to possess not only antimicrobial, anti-inflammatory, and antioxidant properties but also to regulate lipid metabolism, promote growth, and maintain health in animals [193–195]. Their functions in improving chicken IMF content and meat quality have been discovered. The addition of green tea powder (10 g/kg) was noted to positively affect the IMF content, tenderness, and L^*^ and a^*^ values indicative of meat color in breast muscle [196]. Bamboo leaf extract (3 g/kg) was found to be effective in enhancing the WHC, tenderness, and pH_45min_ value of breast meat [197]. Marigold extract (6 g/kg) was observed to bring about improvements in the WHC and tenderness of thigh meat [198]. It has been identified that not all plant extracts are advantageous for meat quality and animal growth. Oregano oil (1 g/kg) was found to exert a positive influence on the pH and WHC of breast meat, but it was concurrently observed to have an adverse effect on meat color and tenderness [199]. Olive pulp (50 g/kg for the starter phase, 80 g/kg for the finisher period) was noted to not only reduce the pH of breast meat but also increase cooking loss and shear force [200]. A combination of turmeric (1%) and black pepper (1%) was found to decrease broiler BWG and FCR, although this combination led to a reduction in AF deposition [201]. Considering the comprehensive effects of plant extracts on texture, meat quality, and broiler growth performance is essential when these extracts are used as supplements.

It has been demonstrated that extracts such as pomegranate and grape seed oil, when used at a concentration of 2%, not only significantly elevated the levels of CLAs and the ratio of ω-6/ω-3 PUFA in muscle tissue, but also notably enhanced the juiciness, tenderness, and flavor of the meat [202]. When *Artemisia afra* (African wormwood) essential oil was supplemented at the 0.1% level, it was found that the IMF content was increased and meat quality traits, such as pH, meat color, WHC, and PUFAs, were improved in Cobb 500 broilers at 35 d. It was also observed that the ω-6/ω-3 PUFA ratio in breast muscle was downregulated and the final body weight of these broilers was increased [203]. These findings suggest the potential of plant extracts to regulate meat quality and fatty acid composition. Given their diversity and multifunctionality, future research should identify their active components and mechanisms, and investigate their metabolic transformation, tissue distribution, and residue levels to ensure their safety and efficacy in poultry breeding and production.

### IMF by nutrigenomics

Advancements in nutrigenomics have confirmed the genetic effects of nutrients on IMF deposition (Table 4). A study on low-protein diets revealed that a diet containing 13.5% CP and ME of 11.75 MJ/kg significantly increased the body fat deposition, including IMF, AF, and hepatic lipids, in Roman chickens at 17 weeks, without impacting their growth performance. Further research indicated that this 13.5% CP diet significantly regulated the expression of lipid metabolism-related genes in the liver, such as *FASN*, *C/EBPβ*, *LPL*, and *CD36*. It also altered the composition of bile acids, including lithocholic acid, ursodesoxycholic acid, and deoxycholic acid, and influenced the cecal microbiota, particularly Bacteroidetes and Firmicutes. These effects were suggested to be partly mediated by the bile acid–microbiota axis, which may influence the regulation of metabolism and gene expression in hosts [204].

**Table 4:** Effects of nutrients on meat quality at the gene and genomic levels

Nutrients of treatments^a^	Breeds	Effects^b^	Pathways^c^	Key genes or proteins^d^	References
13.5% CP with 11.75 MJ/kg ME	Roman	IMF ↑, abdominal fat ↑, and lithocholic acid ↓	FXR–SHP–SREBP1 and PPARγ pathways	*FASN*, *C/EBPβ*, *LPL*, and *CD36*	[204]
Selenomethionine (0.3 mg/kg); *Bacillus subtilis* (10^9^ CFU/kg); or selenomethionine (0.3 mg/kg) + *Bacillus subtilis *(10^9^ CFU/kg)	Ross 308	IMF, WHC, tenderness, meat color, and pH_45min_ ↑	—	Muscle fiber: *Myf5*, *FM*, *MyoG*, *MyoD*, *IGF-1*, *CAPN2*, *CAPN3*, and *SM*; Lipids: *SLC27A1*, *FAT*, *FABP4*, *H-FABP*, *FASN*, and *ACACA*	[205]
Vitamin E (100 IU/kg)	Arbor Acres	IMF ↑	AGE–RAGE, MAPK and FoxO signaling pathways	*ASB2*, *CETP*, *PDK4*, *CYR61*, *AGTR1*, *UCP3*, and *HPGD*	[206]
Vitamin D_3_ (3,750 IU/kg)	Luhua	IMF ↑, abdominal fat ↓, and final body weight ↑	Fatty acid biosynthetic and metabolic pathways	*LPL* and *FATP1*	[207]
Rutin (400 mg/kg)	Qingyuan partridge	IMF and ω-3 PUFAs ↑	AMPK/PPARG	*AMPKα*, *PPARG*, *FADS1*, *ACACA*, *FASN*, and *ELOVL7*	[208]
Chinese Yam polysaccharide (250 or 500 mg/kg)	Crossbred chickens	IMF ↓	Wnt/β-catenin and PPAR	*Wnt1*, *β-catenin*, *PPARG*, and *C/EBPα*	[209]
Fermented citrus pomace (10%)	Qingyuan partridge	IMF, pH_24h_, and meat color b^*^_24h_ value ↑	—	*SREBP1* and *FASN*	[210]
Fresh corn extract (0.6%)	Jingyuan	IMF ↑	Sphingolipid metabolism pathway	*SPHK1*, *CERS1*, *CERS6*, *GLB1L*, *SGMS2*, *UGT8*, and *UGCG*	[211]
Fructose (10%)	Lingshan chicken, yellow-feather chicken	IMF ↑	De novo lipogenesis	ChREBP	[1]
Phosphatidylethanolamine (intramuscular injection, 5 mg/kg or 10 mg/kg)	Sanhuang	Tenderness and meat color a^*^ value ↑	PPAR, terpenoid backbone biosynthesis, cytokine-cytokine receptor interaction, and neuroactive ligand-receptor interaction signaling pathway	*S1PR3*, *FABP4*, *PLIN2*, *APOA1*, *PPARG*, and *CD36*	[212]
C-type natriuretic peptide	S3 strain chickens (a Chinese-developed strain)	IMPs proliferation ↑, IMPs differentiation ↓, and lipolysis ↑	NPRB–cGMP–PPAR pathway	*FABP4*, *FABP5*, *APOA1*, *ACOX2*, *ADIPOQ*, *CD36*, and *LPL*	[213]

^a^The values represent the content or level of addition in the diets, unless otherwise specified. *CP* Crude protein, *CFU* Colony forming unit, *ME* Metabolizable energy

^b^b^*^_24h_The b^*^ value of meat color is measured 24 h after slaughter, *IMF* Intramuscular fat, *IMPs* Intramuscular preadipocytes, *ω-3 PUFAs* Omega-3 polyunsaturated fatty acids, *pH*_*24h*_ The pH of meat is measured 24 h after slaughter, *WHC* Water-holding capacity; ↑: Increased or improved compared to the control group without treatment; ↓: Decreased or diminished compared to the control group without treatment

^c^*AGE–RAGE* Advanced glycation end products–receptor for advanced glycation end products signaling pathway, *AMPK/PPARG* Protein kinase AMP-activated catalytic subunit alpha 1/Peroxisome proliferator activated receptor γ signaling pathway, *FoxO* Forkhead box, sub-group O signaling pathway, *FXR–SHP–SREBP1* Farnesoid X receptor–Small heterodimer partner–Sterol regulatory element-binding protein 1 signaling pathway, *MAPK* Mitogen-activated protein kinase signaling pathway, *NPRB–cGMP–PPAR* Natriuretic peptide receptor B–Cyclic guanosine monophosphate–Peroxisome proliferator activated receptor signaling pathway, *PPAR* Peroxisome proliferator activated receptor signaling pathway, *Wnt/β-catenin* Wingless and Int (Wnt)/β-catenin signaling pathway

^d^Annotations of key genes or proteins in Table 4 can be found in Supplementary Material 2

Heat stress has been proven to negatively impact meat quality. Diets supplemented with selenomethionine, *Bacillus subtilis*, or both were found to increase the IMF content and WHC of muscles in Ross 308 chickens at 42 d. These supplements were also observed to mitigate the adverse effects of heat stress on IMF, WHC, tenderness, meat color, and pH_45min_. This mitigation was identified to be achieved through the regulation of muscle fiber-related genes, such as myogenic factor 5 (*Myf5*), *MyoG*, *MyoD*, insulin-like growth factor 1 (*IGF-1*), *CAPN2*, calpain 3 (*CAPN3*), slow myosin heavy chain (*SM*), and fast myosin heavy chain (*FM*), as well as genes involved in fat synthesis and deposition, such as *SLC27A1*, fatty acid translocase (*FAT*), *FABP4*, *H-FABP*, *FASN*, and *ACACA* [205].

Research on vitamins showed that a diet supplemented with 100 IU/kg of vitamin E enhanced IMF deposition in the breast muscles of Arbor Acres chickens at 35 d. Transcriptome analysis suggested that this enhancement might be attributed to the regulation of signaling pathways, including advanced glycation end products–receptor for advanced glycation end products (AGE–RAGE), MAPK, and FoxO, and associated genes in these pathways, such as ankyrin repeat and SOCS box containing 2 (*ASB2*), *CETP*, pyruvate dehydrogenase kinase 4 (*PDK4*), cysteine-rich angiogenic inducer 61 (*CYR61*), angiotensin II receptor type 1 (*AGTR1*), *UCP3*, and *HPGD* [206]. Another study demonstrated that the IMF content in muscles was significantly increased in Luhua broilers at 84 d when diets were supplemented with 3,750 IU/kg of vitamin D_3_. Gene analysis revealed that this improvement could be linked to the upregulated expression of key genes involved in lipid uptake in muscle, such as *LPL* and *FATP1*. It was also found that this supplementation decreased the AF rate while increasing the final body weight of Luhua broilers [207]. Future studies across different breeds are needed to further validate the potential of this vitamin D_3_ dosage to enhance chicken production.

At the genetic level, the effects of phytochemicals on IMF in chickens have also been confirmed. Dietary supplementation with rutin was reported to enhance the IMF deposition in breast muscles of Qingyuan partridge chickens, particularly at a dosage of 400 mg/kg. This effect was suggested to be attributed to the regulation of the AMPK/PPARγ signaling pathway and the expression of related genes, such as *AMPKα*, *PPARG*, *FADS1*, *ACACA*, *FASN*, and *ELOVL7*. Rutin was also found to increase the levels of ω-3 PUFAs in breast meat [208]. Supplementation of Chinese yam polysaccharide (CYP) at levels of 250 or 500 mg/kg was discovered to significantly reduce the IMF content in the breast muscles of crossbred chickens. This finding was thought to be associated with CYP upregulated the expression of Wnt family member 1 (*Wnt1)* and catenin beta 1 (*β-catenin)* genes, activating the Wingless and Int (Wnt) signaling pathway, and thereby inhibiting the expression of *PPARG* and *C/EBPα* [209]. Supplementation of 10% fermented citrus pomace was reported to promote the lipid synthesis by upregulating the expression of *SREBP1* and *FASN* genes, thereby increasing the IMF content in Qingyuan partridge chickens. The improvement of pH_24h_ and meat color b^*^_24h_ in breast muscle at 124 d was also discovered through this supplementation [210]. A gradient experiment confirmed that a diet supplemented with 0.6% fresh corn extract significantly increased the IMF levels in the breast muscles of Jingyuan chickens. Transcriptomic and metabolomic analyses revealed that several DEGs associated with sphingolipid metabolism, such as sphingosine kinase 1 (*SPHK1*), ceramide synthase 1 (*CERS1*), ceramide synthase 6 (*CERS6*), galactosidase beta 1 like (*GLB1L*), sphingomyelin synthase 2 (*SGMS2*), UDP glycosyltransferase 8 (*UGT8*), and UDP-glucose ceramide glucosyltransferase (*UGCG*), might be involved in the regulation of IMF formation [211].

The regulation of IMF deposition in chickens has also been confirmed by several compounds through various pathways. In Lingshan yellow-feathered chickens, IMF deposition was found to be facilitated following the intake of fructose. Subsequent cellular experiments indicated that fructose could induce the activation of carbohydrate-response element binding protein (ChREBP), thereby upregulating the expression of its downstream genes, such as *THRSP*, *ACACA*, acyl-CoA synthetase short chain family member 1 (*ACSS1*), and *SCD*. This process was identified as activating the ChREBP-mediated DNL pathway, thereby increasing IMF content in chickens. The activation of ChREBP was also found to inhibit the expression of myogenic marker genes, such as *MyoD1*, *MyoG*, and myosin heavy chain, cardiac muscle complex (*MyhC*) [1]. This finding suggests that ChREBP may be a target for regulating muscle development and IMF deposition. Intramuscular injection of phosphatidylethanolamine (PE; 5 mg/kg or 10 mg/kg) was discovered to reduce the shear force of leg muscles and increased the meat color a^*^ value in Sanhuang chickens. Transcriptomic analyses and cellular experiments identified that PE promoted the adipogenic differentiation and lipid accumulation of intramuscular preadipocytes by regulating the expression of key genes associated with classic lipid metabolism pathways, including the PPAR signaling pathway, terpenoid backbone biosynthesis, cytokine–cytokine receptor interaction, and neuroactive ligand-receptor interaction. The identified key regulatory genes include sphingosine-1-phosphate receptor 3 (*S1PR3*), *FABP4*, *PLIN2*, *APOA1*, *PPARG*, and *CD36* [212]. At the cellular level, C-type natriuretic peptide was discovered to regulate the IMF deposition by enhancing the proliferation of intramuscular preadipocytes, but inhibiting their differentiation and promoting lipolysis in chickens via the natriuretic peptide receptor B (NPRB)–cyclic guanosine monophosphate‌ (cGMP)–PPAR pathway. This process was suggested to be mainly achieved by regulating the expression of genes enriched in the PPAR pathway, such as *FABP4*, *FABP5*, *APOA1*, acyl-CoA oxidase 2 (*ACOX2*), *ADIPOQ*, *CD36*, and *LPL* [213]. These studies have established genetic associations between nutrients and the regulation of IMF deposition in chickens from a nutrigenomics perspective, providing practical references for integrating genetic and nutritional strategies to improve meat quality. Future research should delve into the mechanisms of action of more nutrients at the cellular and genetic levels to enhance the understanding of their effects on IMF deposition in chickens, and offer theoretical support for precision nutrition interventions.

### Embryonic nutrition

**Fig. 4:**
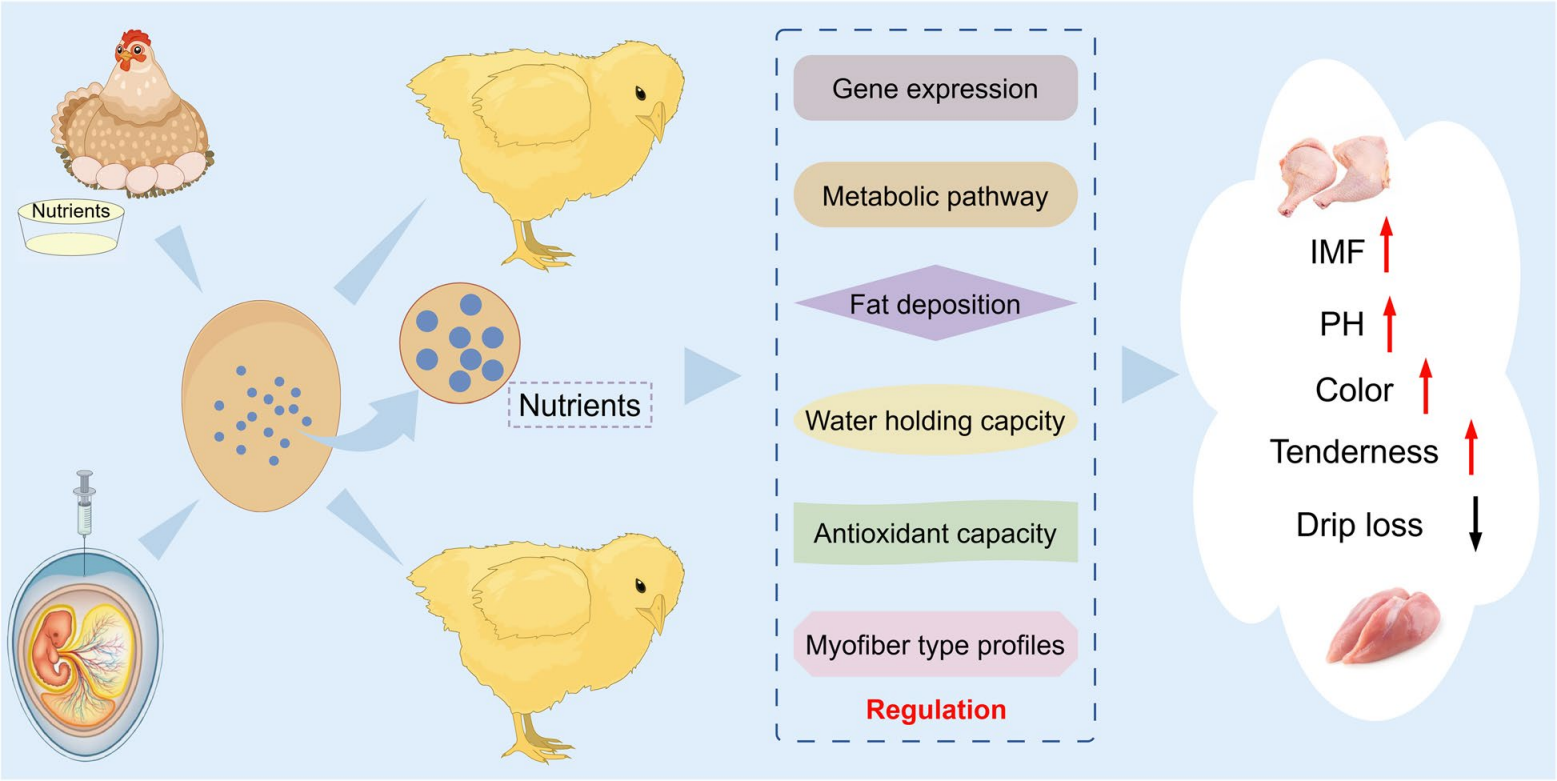
Schematic diagram illustrating the role of enhancing embryonic nutrition in improving the meat quality of broiler chicks. IMF: Intramuscular fat. The red upward arrow represents upregulation, and the black downward arrow represents a decrease. Created by FigDraw (https://www.figdraw.com)

The proliferation of myofibers and intramuscular adipocytes has been characterized primarily to occur during the embryonic phase, and their numbers are essentially established at hatching [50]. This finding indicates the importance of embryonic nutrition for muscle development. Modern fast-growing broilers are typically marketed from 36 to 49 d post-hatch [214]. The 21-d incubation period, which completely relies on the nutrition within eggs, accounts for more than 30% of their life cycle [215]. The effects of maternal nutrition and IOF are becoming a research hotspot, as illustrated in Fig. 4 and Tables 5 and 6.

**Table 5:** Effects of maternal nutrition on offspring growth performance and meat quality

Maternal characteristics or nutrition treatments^a^	Chicken breeds^b^	Main findings of the offspring^c^	References
Breeders with high RMEm	Ross 708	Breast yield ↑, tenderness ↑	[216]
Breeders with high RMEm and low RFI		Best production performance	
Diet, 17.5% CP with 11.51 or 11.92 MJ/kg ME	Chinese yellow feather	Growth performance (starter-phase) ↑	[217]
Diet, 15.5% CP with 11.51 MJ/kg ME		Dressing percentage and meat color (finisher-phase) ↑	
Energy restriction, −2.34 MJ/kg and −3.51 MJ/kg	Arbor Acres	Antioxidant capacity of muscle ↑, IMF ↑, WHC ↑	[218]
Energy restriction from 20% to 50%	Arbor Acres	Myofibers diameter ↓, Myofibers density ↑	[220]
Energy restriction 25%	Fat-line	IMF at 28 d of age ↑, Myofibers diameter ↓, Myofibers density ↑	[219]
	Lean-line	Myofibers diameter↑, Myofibers density ↓	
Diets with 1% coated-Met	Ross	Final body weight ↑, dressing percentage ↑, and muscle weight ↑, breast muscle pH_45min_ and tenderness ↑, meat color a^*^ value ↓	[223]
Paternal diet with 1% coated-Met	Ross	Dressing percentage ↑, Thigh muscle ↑, pH ↑, WHC ↑, tenderness ↑, meat color L^*^ and a^*^ values ↑	[224]
Diets with 100 mg/kg of zinc sources	Ross 308	Breast muscle percentage (finisher phase) ↑	[225]
Diets with 50 mg/kg or 300 mg/kg of ZnSO_4_	Ross 308	Myofiber width (starter-phase) ↑	[226]
Diets with 500 mg/kg of collagen peptide chelated trace elements	Qiling	Breast muscle percentage ↑	[227]
Diets with 0.3 mg/kg of organic selenium	Lingnan-yellow and Ross-308	WHC and antioxidant capacity ↑	[228, 229]
Diets with 80 mg/kg of zinc, either as ZnSO_4_ or Zn-Gly	Lingnan Yellow	Antioxidant capacity ↑ oxidative stress↓	[231]
Diet with 21,600 IU/kg of vitamin A	Chinese yellow-feathered	pH_24h_ ↑	[234]
Vitamin E combination with soybean oil	Ross	Breast muscle color reduced linearly	[235]
Vitamin E combination with fish oil		pH_24h_ of breast muscle increased linearly, 187 mg/kg of Vitamin E resulted in the lowest drip loss	
Co-supplementation 100 mg/kg vitamin E in maternal and offspring diets	Broiler (breed unspecified)	WHC ↑, antioxidant capacity ↓	[236]
Diets with 40 mg/kg of GEN	Ross 308	Embryonic length, weight, and width of the proliferative zone in the tibial growth plate ↑	[238]
Maternal diet with 400 mg/kg of GEN + Offsprings’ diet with 40 mg/kg of GEN	Ross 308	BWG and breast or leg muscle rate (psot-hatch) ↑	[239]
Maternal diet with 20 mg/kg of GEN	Qiling chickens	BWG↑, morphology of intestinal villi ↑, structure of the gut microbiota ↑, intestinal mucosal injury ↑	[240]
Maternal diet with 60 mg/kg of HLF	Qiling chickens	BWG ↑, Intestinal development ↑, microbial community structure ↑	[241]
Maternal diet with 30 or 60 mg/kg of MLF	Qiling chickens	BWG ↑, breast muscle rate ↑, cross-sectional area of breast myofibers ↑	[242]

^a^The values represent the content or level of addition in maternal diets, unless otherwise specified; *CP* Crude protein, *GEN* Genistein, *HLF* Hawthorn-leaf flavonoids, *ME* Metabolizable energy, *Met* Methionine, *MLF* Mulberry-leaf flavonoids, *RFI* Residual feed intake, *RMEm* Residual maintenance requirement, *Zn-Gly* Zinc glycine, *ZnSO*_*4*_ Zinc sulfate; + : And

^b^Fat-line and lean-line: Two lines of broiler breeders developed at Northeast Agricultural University in China

^c^*BWG* Body weight gain, *IMF* Intramuscular fat, *pH*_*24h*_ The pH of meat is measured 24 h after slaughter, *WHC* Water-holding capacity; ↑: Increased or improved compared with the control group without treatment; ↓: Decreased or diminished compared with the control group without treatment

**Table 6:** Effects of IOF on muscle development and meat quality in broiler chickens^a^

Nutrient^b^	Chicken breeds	Injection dosage, /egg^c^	Time^d^	Effects^e^	References
N-carbamylglutamate	Ross 308	2 mg	17.5ED	Type I myofibers ↑ IMF, WHC, tenderness ↑ Antioxidant capacity ↑	[244, 245]
Inulin + *Lactococcus lactis* subsp. *lactis*	Ross 308	1.760 mg inulin and 1,000 CFU *Lactococcus lactis* subsp*. lactis*	12ED	Type I myofibers ↓ Type II myofibers ↑	[246]
Vitamin C	Ross 708	36 mg	17ED	Tenderness ↑	[247]
	Cobb 500	6 μg	18ED	Toughness of breast meat ↓	[248]
	Arbor Acres	3 mg	15ED	Antioxidant capacity ↑	[247, 249]
	Ross 708	12 mg	17ED		
Vitamin E	Cobb 500	60.4 IU	17.5ED		[250, 251]
Canthaxanthin	Cobb 500	0.65 mg	17.5ED		
25-hydroxyvitamin D_3_	Ross 708	2.4 μg	18ED	Incidence of wooden breast ↓	[252, 253]
Equol	Sanhuang	100 μg	7ED	Drip loss ↓ Meat color a^*^ and b^*^ values ↓ Tenderness ↑	[254]
*Lactobacillus plantarum* + Lupin RFO	Cobb 500	2 mg of raffinose family oligosaccharides, enriched with 10^5^ bacteria CFU of *Lactobacillus plantarum*	12ED	IMF ↓ meat color L^*^_45min_ value ↓	[256]
Zinc	Ross 308	60 mg	18ED	pH ↑	[257]
Galactooligosaccharides or sodium butyrate	Ross 308	Galactooligosaccharides (3.5 mg); sodium butyrate (0.6 mg)	12ED	IMF↓ WHC↑ (leg muscles)	[258]

^a^*IOF* In ovo feeding

^b^*RFO* Raffinose family oligosaccharides

^c^*CFU* Colony forming unit

^d^*ED* Embryonic day

^e^*IMF* Intramuscular fat, *WHC* Water-holding capacity, L^*^_45min_ The L^*^ value of meat color is measured 45 min after slaughter; ↑: Increased or improved compared with the control group without treatment; ↓: Decreased or diminished compared with the control group without treatment

#### Maternal nutrition

Maternal nutrient intake and energy metabolism have been recognized as having direct impacts on offspring growth performance and meat quality (Table 5). It has been demonstrated that the offsprings from breeders with high residual maintenance requirement (RME_m_) exhibit greater breast yield (29.5%) and tenderness (4.7% lower force/g) compared to those from breeders with low RME_m_. Offsprings’ growth and development performance have also been observed to be inversely correlated with the residual feed intake (RFI) of their mothers. The optimal body weight gains at market age were observed in the offsprings from mothers with low RFI and high RME_m_ [216]. Specific maternal diets, such as 17.5% CP with 11.51 or 11.92 MJ/kg ME, have been discovered to enhance the growth performance of offsprings during the starter phase, while 15.5% CP with 11.51 MJ/kg ME has been noted to enhance dressing percentage and meat color during the finisher phase [217]. In Arbor Acres broilers, it has been found that moderate maternal energy restriction (−2.34 MJ/kg and −3.51 MJ/kg) could enhance the muscular antioxidant capacity of the offsprings, but excessive restriction (−5.85 MJ/kg) might be harmful [218]. Maternal energy restriction has been reported to increase the IMF content in offspring breast muscle at 28 d of age [218, 219]. These effects did not persist into the finisher stage, which suggests that the maternal effects are more significant during the early growth stage. It was also reported that maternal energy restriction improved the WHC of muscle at market age in Arbor Acres broilers [218].

Maternal energy restriction has been found to influence the phenotypic characteristics of myofibers of offsprings (Table 5). In Arbor Acres broilers, an increase in maternal energy restriction has been detected to be associated with a decrease in the diameter of breast myofibers in the offsprings, while their density has increased [220]. Similar findings have also been observed in fat-line broilers [219]. The results in lean-line broilers have been shown to be opposite, which could imply an interaction between maternal energy intake and broiler strains [219]. Broiler breeders of different strains have been found to have varying energy requirements for their own maintenance and the embryonic development of their offsprings. The different results observed in fat-line broilers could be attributed to the fact that undernutrition leads to insufficient myofiber development [221, 222]. Future research should aim to clarify the regulatory mechanisms underlying this interaction and its impact on the meat quality of offsprings.

The growth performance and meat quality of offsprings have been discovered to be regulated by maternal Met intake (Table 5). It has been shown that a maternal diet supplemented with 1% coated-Met increased the final body weight, dressing percentage, and muscle weight of the offsprings, as well as improved the pH_45min_ and tenderness of the breast muscle, along with decreasing the meat color a^*^ value. Excessive Met supplementation, such as 2%, has been shown to be harmful to the growth performance of the offsprings [223]. Supplementation of the paternal diet with 1% coated-Met has also been shown to have effects similar to those of the maternal diet, which increased the WHC and meat color L^*^ and a^*^ values in the offspring’s muscles [224]. These results emphasize the importance of parental amino acid nutrition for muscle development and meat quality in offsprings.

The supplementation of maternal trace elements has been shown to promote muscle development in offsprings (Table 5). An increase in the breast muscle percentage of offspring chicks during the finisher phase has been reported when the maternal diet was supplemented with 100 mg/kg zinc sources, such as zinc sulfate (ZnSO_4_) or methionine hydroxy analog-chelated zinc [225]. Their myofiber width was detected to be significantly increased when maternal diets were supplemented with 50 mg/kg or 300 mg/kg of ZnSO_4_ [226]. The enhancement of muscle development in the offsprings has been proposed to be attributed to the detected deposition of zinc in hatching eggs, which promoted protein synthesis by activating the AKT/mTOR signaling pathway and reduced protein degradation by inhibiting the ubiquitin‒proteasome system pathway [225, 226]. A recent study has indicated that the deposition of Fe, Mn, and Zn in the yolks of hatching eggs was increased to some extent by supplementing the maternal diet with 500 mg/kg of collagen peptide chelated trace elements, providing 11.65 mg/kg Fe, 8.1 mg/kg Mn, and 32.88 mg/kg Zn. An increase in the breast muscle rate of the offsprings was observed due to this supplementation, which promoted protein metabolism and upregulated the expression of myogenic factors by activating the IGF-1 signaling pathway [227]. These findings provide a theoretical basis for improving muscle development in offsprings through maternal trace element supplementation.

The meat quality of offsprings has also been shown to be influenced by the sources of trace elements in maternal intake (Table 5). It was shown that the WHC and antioxidant capacity of the breast muscle of offsprings were significantly enhanced by supplementing the diets of Lingnan-yellow chickens (an indigenous Chinese breed) and Ross 308 broiler breeders with 0.3 mg/kg of organic selenium, in the form of selenomethionine, selenium-enriched yeast, or hydroxy-selenomethionine [228, 229]. This significant effect was not observed in Ross-308 broilers during the finisher phase [228], suggesting that there might be an interaction between the supplementation of maternal trace elements and chicken breeds. The interaction may be attributed to variations in the genetic structure among different chicken breeds. Chinese local chicken breeds, which are widely recognized as typically having a greater proportion of oxidative myofibers and better meat quality [230], might be more sensitive to trace element metabolism. This characteristic could lead to the carry-over of maternal effects into the finisher phase. Future research needs to delve into this interaction and explore the underlying mechanisms affecting the persistence of maternal effects.

Maternal diets supplemented with 80 mg/kg zinc, in the form of either ZnSO_4_ or zinc glycine (Zn-Gly), have been discovered to enhance antioxidant capacity and alleviate oxidative stress in the offsprings, thereby reducing embryo mortality under heat stress (Table 5). The observed improvement has been confirmed to be attributed to the activation of the nuclear factor erythroid 2-related factor 2 (Nrf2) signaling pathway and the reduction in CASP3 activity, both of which have been identified to slow down oxidative stress injury and tissue cell apoptosis [231]. Organic zinc in maternal diets was found to exert more pronounced effects on offsprings than inorganic zinc [231, 232], and it was found to improve the b^*^ value of meat color [232]. Maternal supplementation with a combination of organic trace elements, including Fe, Cu, Mn, and Zn, was observed to increase body weight and FCR in the offsprings during the starter phase [233]. Further research is needed to understand the impact of trace element supplementation in maternal diets on IMF deposition in the offsprings.

Regarding vitamins, it has been demonstrated that maternal diets supplemented with vitamin A have a relatively limited impact when compared to feeding the offspring directly (Table 5). It was reported that a maternal diet supplemented with 21,600 IU/kg of vitamin A only improved the pH_24h_ in the breast muscle of offsprings at market age. However, feeding offsprings a 5,000 IU/kg vitamin A diet directly was found to not only significantly improve the pH_45min_ and tenderness of the breast muscle but also enhanced the WHC and meat color, specifically the L^*^ and b^*^ values [234]. These findings suggest that the effects of vitamin A supplementation in maternal diets on the offsprings’ meat quality might be limited.

Some studies have shown that the interaction between maternal dietary vitamin E levels and oil sources significantly affected offsprings’ meat quality (Table 5). When soybean oil was the source, maternal vitamin E supplementation linearly reduced the breast muscle color; when fish oil was used, the same vitamin E treatment linearly increased the pH_24h_ of the breast muscle, and at a dose of 187 mg/kg, it resulted in the lowest drip loss [235]. These results reveal the oil source-dependent effects of maternal dietary vitamin E on the meat quality of offsprings. Although co-supplementation with 100 mg/kg vitamin E in both maternal and offspring diets reduced the drip loss of breast meat, it also impaired the antioxidant capacity of offsprings [236]. Long-term high-dose vitamin E supplementation may lead to its accumulation, which can produce additive effects and thereby promote the occurrence of peroxidation in the offsprings [237]. These findings emphasize the necessity of balancing vitamin E intake between mother and offsprings. Further research is needed to determine the optimal dosage of vitamin E supplementation for breeders and to elucidate the specific mechanisms by which it interacts with oils to regulate the meat quality of offspring broilers.

Several specific phytochemicals that are supplemented in maternal diets have been proven to promote muscle development and growth performance in offsprings (Table 5). For instance, supplementing maternal diets with genistein (GEN, 40 mg/kg) was shown to increase embryonic length and weight, as well as the width of the proliferative zone in the tibial growth plate of offspring embryos. Its underlying mechanisms were demonstrated to involve the regulation of insulin-like growth factor-binding protein 3 (*IGFBP3*) gene expression, the activation of glycolipid metabolism pathways, and the enhancement of antioxidant capacity and immune function [238]. Maternal supplementation with GEN was found to improve body weight gain and the rate of breast or leg muscle development in offspring post-hatch, while also optimizing the morphology of the intestinal villi and the structure of the gut microbiota, thereby alleviating mucosal injury [239, 240]. A maternal diet supplemented with hawthorn-leaf flavonoids (60 mg/kg) was found to exert similar effects on intestinal development and microbial community structure and to enhance offsprings’ growth performance via the IGF-1R/AKT/mTOR signaling pathway [241]. Maternal intake of mulberry-leaf flavonoids (MLF, 30 or 60 mg/kg) was observed to increase body weight gain and the breast muscle rate and to enlarge the cross-sectional area of breast myofibers in the offsprings [242]. These benefits have been suggested to be attributed to the upregulation of *Myf5* gene expression and the activation of the Bone Morphogenetic Proteins (BMP)/p-SMAD1/5/9 axis, both of which collectively promote muscle development. Maternal intake of soyasaponin (200 mg/kg) was discovered to interact with vertically transmitted *Bifidobacterium adolescentis* to produce γ‐aminobutyric acid (GABA). GABA was subsequently identified to modulate offspring intestinal development by inhibiting autophagy and apoptosis pathways associated with microtubule-associated protein 1 light chain 3 (LC3) and CASP3, as well as by promoting proliferation and differentiation pathways mediated by proteins, leucine rich repeat containing G protein-coupled receptor 5 (LGR5) and olfactomedin 4 (OLFM4) [243]. These findings confirm that maternal intake of phytochemicals can positively regulate the growth and development of offsprings by modulating growth pathways and improving gut health. They also imply that maternal phytochemical intake might influence the IMF content and meat quality in the offsprings, warranting further investigation.

In chickens, the influence of maternal fatty acid supplementation on offsprings’ meat quality has not yet been fully explored. Those studies focused primarily on the effects of PUFA supplementation on fatty acid composition of eggs, as well as on growth performance, immune response, and central nervous system development of the offsprings [214]. Fatty acids have been acknowledged as crucial nutrients for embryonic development, providing both energy and functional fatty acids such as essential ω-6 and ω-3 PUFAs. Specific long-chain fatty acids, such as oleic acid, palmitic acid, and arachidonic acid, have been demonstrated to promote the proliferation of MuSCs [171], whereas DHA has been identified to promote adipocyte production and lipogenesis by activating the free fatty acid receptor 4 (FFAR4) in FAPs [172]. Considering the development characteristics of myofibers and adipocytes during the embryonic period, maternal fatty acid supplementation could significantly affect the IMF content and meat quality of offsprings. Future research should focus on the regulatory effects and underlying mechanisms of fatty acid types, sources, and dosages in maternal diets on the IMF content and meat quality of offspring.

#### In ovo feeding

IOF has been advocated as a compensation strategy for embryonic nutritional deficiencies to ensure muscle development and meat quality in chicks, and has garnered increasing attention from researchers (Table 6). Injecting nutrients such as synbiotics, vitamins, trace elements, amino acids, and hormone analogs into eggs has been reported to improve broiler meat quality through multiple biological mechanisms. It was found that the percentage of type I myofibers in broiler chicks was significantly increased by the IOF of N-carbamylglutamate (NCG, 2 mg/egg) at 17.5 ED, resulting in enhanced the IMF content, antioxidant capacity, WHC, and tenderness of the breast muscle at market age. This improvement has been demonstrated to be associated with NCG enhancing the gene expression of *MSTN* and *PGC-1α*, which in turn activates the gene expression of *PPARG* and regulating myofiber types [244, 245]. When a combination of inulin and *Lactococcus lactis* subsp*. lactis* was employed for IOF at 12ED, a significant reduction in the proportion of type I myofibers and increase type II myofibers at 35 d in Ross 308 broilers were observed [246]. These findings indicate that meat quality can be regulated by IOF via its effects on myofiber types.

Vitamins administered through IOF have been found to improve the quality of chicken meat (Table 6). It was reported that an IOF of 36 mg/egg of vitamin C at 17ED significantly improved the tenderness of breast meat at market age [247]. An IOF of a low dose of vitamin C (6 μg/egg) at 18ED was shown to effectively prevent the toughness of breast meat caused by heat stress during incubation [248]. The IOF of vitamin C at 3 mg/egg at 15ED and at 12 mg/egg at 17ED was observed to enhance the antioxidant performance of broilers post-hatch [247, 249]. An IOF of 60.4 IU/egg of vitamin E or 0.65 mg/egg of canthaxanthin at 17.5ED was discovered to exhibit similar antioxidant effects [250, 251]. These results emphasize the beneficial impacts of IOF of vitamin C and vitamin E on the taste and antioxidant capacity of chicken meat.

The IOF of vitamins has been reported to help reduce the occurrence of wooden breast in broilers (Table 6). Studies showed that the incidence of wooden breast in broilers at market age decreased after the IOF of 25-hydroxyvitamin D_3_ (25-OHD_3_) was applied at 18 ED. This effect might be attributed to the long half-life of 25-OHD_3_, which remains in the muscle tissue for a longer period, thereby potentially alleviating muscle inflammation related to the development of wooden breast [252, 253]. Studies have shown that the effects of IOF on chicken meat quality can vary significantly between genders. In female broilers, a significant decrease in drip loss and meat color, specifically the a^*^ and b^*^ values, and an improvement in tenderness at market age were observed after an IOF of 100 μg/egg equol was applied at 7 ED [254]. However, similar results have not been observed in males. These distinct results may be attributed to the gender-specific susceptibility and hormonal responsiveness in broiler chickens, which could explain the observed differences in meat quality. Similar evidence has also been found in another study, which demonstrated that testosterone could promote muscle growth and reduce IMF deposition in roosters but not in hens [255]. These findings underscore the importance of considering the interactions between nutrients and gender when IOF strategies are applied. A deeper investigation into the mechanisms underlying these interactions, particularly in the regulation of IMF and meat quality, would be beneficial for the refinement and optimization of these strategies.

Recently, a study found that IOF of synbiotics (*Lactobacillus plantarum*+Lupin raffinose family oligosaccharides) at 12ED significantly reduced the IMF content and meat color L^*^_45min_ values in the breast muscles of broilers at market age [256]. The IOF of zinc (60 mg/egg) at 18ED was observed to significantly increase the pH of breast meat at market age, which was superior to that of the control group and the experimental groups fed with zinc only after hatching (100 mg/kg or 200 mg/kg; see Table 6) [257]. The IOF of galactooligosaccharides (3.5 mg/egg) or sodium butyrate (0.6 mg/egg) at 12ED was reported to significantly reduce the IMF content in muscles, but improve the WHC of leg muscles in Ross 308 chickens at 42 d [258]. These findings further confirm the regulatory effects of IOF on IMF and meat quality, and indicate that the administration of certain substances via IOF may not be conducive to IMF deposition in chickens. Current research on embryonic nutrition primarily focuses on the phenotypic characteristics of growth performance and meat quality, but has limited exploration of both IMF deposition and the regulatory mechanisms underlying these phenotypes. Future research should aim to deepen the understanding of these regulatory mechanisms.

## Signaling pathways in MSCs for adipogenic commitment

The classical theory suggests that myoblasts, intramuscular adipocytes, and osteocytes all originate from paraxial MSCs during embryonic development. These cells are committed to differentiating into satellite cells, FAPs, or osteoblasts under the precise and complex regulation of signaling pathway networks [14–17, 259]. These related pathways, including Wnt/β-catenin, AMPK, Sonic Hedgehog (SHH), TGF-β/SMAD, BMP, Actin and Ras Homologous (Rho) signaling, have been identified to play important roles in the adipogenic commitment of MSCs. Du et al. [260] and Liu et al. [261] have provided extensive reviews on these topics. Here, we focus on the Wnt/β-catenin, AMPK, and SHH signaling pathways.

### Wnt/β-catenin signaling

Wnt signaling pathway is highly conserved and widely present in multicellular organisms. The canonical Wnt pathway relies on β-catenin and is commonly referred to as the Wnt/β-catenin signaling pathway [260, 262]. In skeletal muscle, β-catenin has been identified to interact with several transcription factors to regulate the expression of its downstream genes, thereby promoting muscle development. *Myf5* is a direct target of the Wnt/β-catenin pathway [263]. The Wnt signaling pathway has been found to act as a molecular switch to balance myogenesis and adipogenesis. When the Wnt signaling pathway is activated, the myogenesis of MSCs is promoted, while their adipogenesis is inhibited by downregulating the expression of *PPARG* and *C/EBPα* [264, 265]. Conversely, blocking the β-catenin pathway has been reported to reduce the myogenesis process [266, 267]. A study has shown that the Wnt/β-catenin signaling pathway is associated with the IMF content in the longissimus dorsi muscle of cattle. The enhancement of Wnt signaling downregulates the expression of *PPARG* and *C/EBPα* genes, thereby decreasing the formation of IMF. In contrast, the downregulation of β-catenin has been found to promote the expression of *PPARG* and adipogenic differentiation of bone marrow-derived MSCs [268]. The results mentioned in Sect. "IMF by nutrigenomics" on the effects of CYP dietary supplementation also confirm the regulatory role of this pathway in chicken IMF deposition. These results suggest that the function of CYP may be achieved by regulating the differentiation of MSCs [209]. In Sect. "Epigenetic modifications", which discusses the regulation of IMF by DNA methylation, researchers have reported that some DMGs are enriched in the Wnt signaling pathway, although these specific DMGs were not listed [70]. These results confirm that the Wnt/β-catenin signaling pathway is highly likely to play a switch-like role in regulating the commitment of MSCs to preadipocytes during embryonic development in chickens. Further in-depth studies of this pathway are expected to enable precise regulation of IMF formation at the stem cell level.

### AMPK signaling

AMPK is an intracellular kinase that regulates adipogenesis by promoting various biological processes, such as fatty acid oxidation and synthesis [269]. Its activity has been found to be negatively correlated with the IMF content in cattle but positively correlated with the degree of muscle development [270]. AMPK is suggested to regulate the differentiation of MSCs in skeletal muscle, switching MSCs from adipogenesis to myogenesis. For example, activation of AMPK by AICAR increased the expression of myogenic enhancer factor-2 (*MEF2*), thereby enhancing myogenesis [271]. Its activation also promoted fatty acid oxidation by phosphorylating ACC2, thereby reducing TG synthesis [272]. Such activation has been shown to regulate lipid aggregation in myocytes by modulating the m^6^A demethylation of mRNA through the *FTO* gene [273]. It downregulated adipogenic markers, such as *PPARG*, *C/EBPα*, and *FABP4*, thereby inhibiting adipogenesis [274]. Activation of AMPK by sirtuin 5 (SIRT5) has been reported to inhibit the MAPK pathway, thereby suppressing the differentiation and lipid deposition of bovine preadipocytes [275]. In Sect. "LncRNAs and circRNAs", which discusses ncRNAs, the regulatory role of AMPK in chicken muscle development has also been confirmed [81, 111]. In Sect. "IMF by nutrigenomics", it is reported that the supplementation of rutin inhibits the expression of the *AMPKα* gene and increases the IMF content in chickens [208]. This finding further suggests that the AMPK signaling pathway may inhibit the commitment of MSCs to preadipocytes in chickens. AMPK signaling pathway is likely to be another important target for precisely regulating IMF formation in chickens, which needs to be further confirmed at the stem cell level.

### SHH signaling

A rising number of studies have revealed the effects of SHH signaling in adipogenesis and lipogenesis. For example, the activation of the SHH signaling pathway was discovered to inhibit adipogenesis in the pluripotent mouse embryonic fibroblast cell lines, 3 T3-L1 and C3H10T1/2 [276–279]. The reduction of SHH signaling was suggested to be necessary but not sufficient to trigger adipocyte differentiation [280]. It was thought that SHH signaling promotes stem cell differentiation into either myogenic or osteogenic cells, while the down-regulation of SHH enhances adipogenesis [260]. This signaling pathway has been demonstrated to resist adipogenesis by promoting the expression of GATA family member 2 (*GATA2*), which directly interacts with C/EBPα and PPARγ, thereby preventing PPARγ from exerting its pro-adipogenic effects [281, 282]. The functions of GATA proteins include promoting myogenesis and other cell differentiations by recruiting MEF2 [283]. It was also demonstrated that the SHH signaling negatively regulates adipogenesis through nuclear receptor subfamily member 2 (NR2F2), which binds to the promoter regions of *C/EBPα* and *PPARG* to inhibit their expression [284]. The role of SHH signaling in the commitment of MSCs to the adipogenic lineage in chicken needs further investigation.

These findings have demonstrated that PPARγ is regulated by multiple signaling pathways and acts as a key downstream factor in the adipogenic commitment of MSCs. The ncRNAs, such as lncRNA *IMFNCR*, miR-27b-3p, and miR-128-3p, mentioned in Sect. "LncRNAs and circRNAs", have been identified as regulators of *PPARG* function and the adipogenic process in chickens [94–96]. This suggests that these ncRNAs may play a central role in the adipogenic commitment of MSCs and the muscle–adipose balance in chickens. Further investigation at the stem cell level is needed to elucidate the adipogenic differentiation fate of chicken MSCs, building on the groundwork laid by these findings.

### Exosomes involved in muscle–adipose interactions

Exosomes are small vesicles with a lipid bilayer membrane structure secreted by cells. As important carriers for intercellular communications, they have been found to regulate both systemic metabolism and the physiological responses of target cells by carrying and transferring substances, such as miRNA, lncRNA, mRNA, cytokines, enzymes, and lipids [285, 286]. They are increasingly recognized as mediators of interactions between muscle and adipose tissues. A study showed that exosomes secreted by adipose-derived MSCs (AD-MSCs) upregulate the expression of myogenic genes, including actin alpha 1, skeletal muscle (*ACTA1*), dystroglycan 1 (*DAG1*), desmin (*DES*), troponin T1, slow skeletal type (*TNNT1*), and myosin heavy chain 1 (*MYH1/2*). When skeletal muscles were injured, these exosomes were found to enrich growth factors, which stimulate the differentiation of AD-MSCs into the myogenic lineage and thereby activate muscle regeneration [287]. In sarcopenic mice, adipose-derived exosomes were found to carry miRNA Let-7d-3p, which inhibits the expression of high mobility group AT-hook 2 (*HMGA2*) and thus reduces the proliferation of MuSCs [288]. A recent study in mice showed that muscle injury activates FAPs, which temporarily increase intramuscular adipose tissue (IMAT). These FAPs were found to release extracellular vesicles rich in specific miR-127-3p, which promotes myogenesis by targeting sphingosine-1-phosphate receptor 3 (*S1PR3*) in MuSCs. In contrast, vesicles released by muscle cells, enriched in miR-206-3p and miR-27a/b-3p, inhibit the adipogenesis of FAPs by targeting *PPARG*, thereby reducing IMAT accumulation [289]. In chickens, there is currently only one study on the relationship between exosomes and IMF formation, which was mentioned in Sect. "LncRNAs and circRNAs". This study identified that *LncHLFF* is delivered into intramuscular preadipocytes by hepatocyte-derived exosomes via blood circulation to promote IMF deposition [99]. As exosome research emerges, exploring its role in muscle–adipose tissue interactions in chickens will deepen our understanding of the mechanisms underlying meat quality regulation. Future studies should focus on the roles of exosome-carried cytokines, noncoding RNAs, and metabolites in these interactions to expand our knowledge. 

## Conclusions

**Fig. 5:**
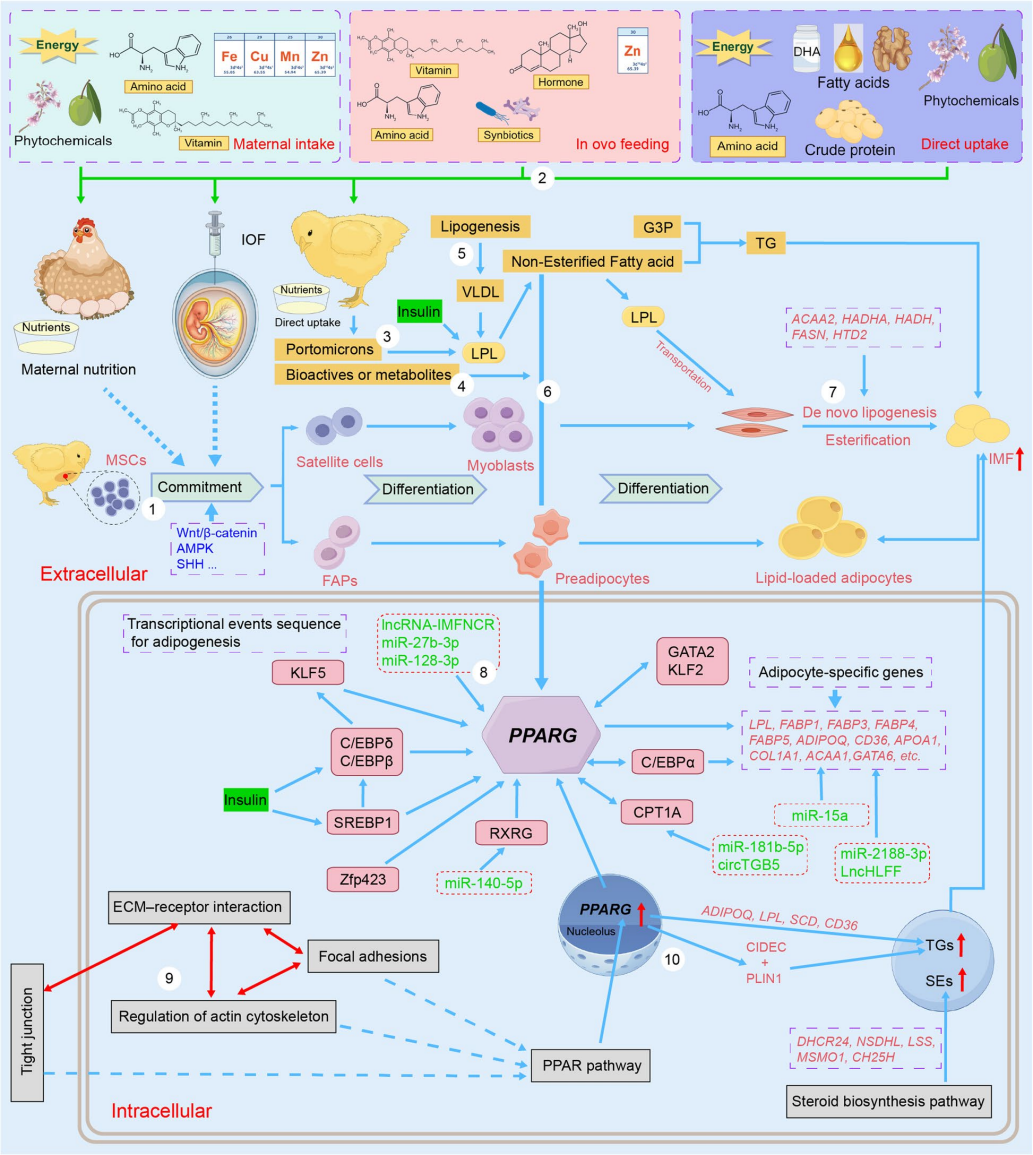
Schematic representation of key genetic pathways in the gene-nutrition network regulating intramuscular fat (IMF). A range of regulatory factors collectively influence IMF formation and deposition in chickens. These factors include maternal nutrition, in ovo feeding (IOF), and direct uptake, as well as biological regulators such as hormones (green boxes), key transcription factors (pink boxes), and non-coding RNAs (ncRNAs; red dashed boxes), along with their target genes and associated pathways. All these elements interact to orchestrate the processes of IMF generation and accumulation. Blue dashed arrows indicate possible regulatory relationships, blue solid arrows indicate regulation, transport, or interaction, and red double-sided arrows indicate bidirectional regulatory relationships. The red upward arrow represents an increase. Figure 5 is based on the reports from [298] and [39], with some modifications. Additional annotations can be found in Supplementary Material 3. Created by FigDraw (https://www.figdraw.com)

This review has comprehensively summarized the research progress in genetic and nutritional regulation of IMF deposition and meat quality in chickens (Fig. 5). scRNA-seq has uncovered that DNL predominantly occurs in myocytes, which is key to the formation of IMF in chicken muscle tissue. Fatty acid synthase (FASN) is the key enzyme involved in this process. This finding reshapes the conventional understanding and offers a fresh perspective on intramuscular lipid metabolism.

Key genes, proteins, and pathways, such as *FASN*, *FABP4*, *PPARG*, *C/EBPα*, *SLC27A1*; LPL, APOA1, COL1A1; PPAR and ECM–receptor interactions signaling, have been identified (Tables 1 and 7). These elements influence IMF content and distribution by regulating fatty acid metabolism and adipogenesis. LncRNA *LncHLFF* promotes ectopic IMF deposition in chickens via exosome-mediated mechanisms without affecting AF deposition, marking an innovative discovery for targeted IMF regulation. The regulations of other ncRNAs in IMF deposition have also been discussed. For instance, miR-27b-3p inhibits adipogenic differentiation by targeting *PPARG*, and miR-128-3p affects IMF formation by regulating *FDPS* and *PPARG*.

**Table 7:** Key findings in the genetic regulation of IMF deposition in chickens

Key findings	Annotations	References
Highlights^a^		
DNL in myocytes	Single-cell RNA sequencing confirmed that DNL mainly occurs in myocytes, contributing to IMF deposition, revealing novel insights	[65]
* LncHLFF*	Promoting ectopic IMF deposition via exosome-mediated mechanisms without affecting AF deposition	[99]
LncRNA *IMFNCR*	Regulating miR-128-3p and miR-27b-3p through the ceRNA mechanism, mediating the muscle–adipose balance in chickens	[94–96]
ChREBP	ChREBP was confirmed to be activated by fructose, thereby stimulating the muscle DNL pathway and inhibiting myogenesis	[1]
Key genes^b^		
* FABP4* and *H-FABP*	Positively associated with IMF content and influenced by chicken gender	[26, 28, 39, 42, 46, 205, 212, 213]
* SLC27A1* (*FATP1*)	Regulating the uptake of saturated fatty acids into myoblasts to reduce CPT1 A-mediated fatty acid oxidation, thereby promoting IMF deposition	[31, 56, 205, 207]
* PPARG* and *C/EBPα/β*	Promoting intramuscular adipogenic differentiation	[39, 44, 69, 204, 208, 209, 212]
* FASN*, *SREBP1*, *SCD*	Involving lipid synthesis	[39, 42, 43, 65, 204, 206, 208, 210]
* PLIN2*	Promoting IMF deposition	[56, 59, 212]
* APOA1*, *COL1A1*, and *ADIPOQ*	Potential biomarkers for IMF	[39, 41, 49, 52, 64, 212, 213]
* TMEM164*	Mainly involving the positive regulation of IMF deposition and having a certain negative regulatory effect on AF deposition	[48]
* G0S2*	Promoting the differentiation of intramuscular preadipocytes and IMF deposition by regulating *SCD* and its downstream genes	[42, 43]
* STC2*	Altering the ratios of long-chain unsaturated fatty acids and glycerophospholipids	[45]
* TIMP2*	Promoting IMF deposition through the ECM–receptor interactions signaling pathway	[68]
* IGFBP7*	Promoting the proliferation and differentiation of primary myoblasts and intramuscular preadipocytes	[69]
Key pathways^c^		
PPAR pathway	Regulating adipocyte differentiation and lipid metabolism-related gene expression to promote IMF deposition and storage	[29, 30, 39, 41, 43, 46, 50, 52, 53, 55, 56, 59, 64, 71, 204, 208, 209, 212, 213]
ECM–receptor interactions	Regulating extracellular matrix-cell signaling to influence adipocyte adhesion and localization, maintaining tissue structural integrity	[29, 32, 41, 46, 52, 64, 68, 70]
Focal adhesion	Mediating mechanical connections between adipocytes and the matrix, modulating intracellular signaling to regulate fat deposition dynamics	[29, 41, 46, 52, 64, 70]
Cell adhesion molecules	Facilitating adipocyte migration and aggregation through cell–cell interactions, influencing IMF distribution	[41, 53, 74]
Wnt/β-catenin	Inhibiting preadipocyte differentiation into mature adipocytes, suppressing IMF formation and maintaining metabolic homeostasis	[64, 70, 209, 263–268]
AMPK	Activating fatty acid oxidation and energy expenditure while inhibiting lipogenesis, thereby reducing IMF accumulation	[81, 111, 208, 269, 270, 272–275]

^a^*AF* Abdominal fat, *ceRNA* Competing endogenous RNA, *ChREBP* Carbohydrate-response element binding protein, *DNL* de novo lipogenesis, *IMF* Intramuscular fat, *LncRNA/Lnc* Long noncoding RNA, *miR* MicroRNA, *scRNA-seq* Single-cell RNA sequencing

^b^*APOA1* Apolipoprotein A1, *ADIPOQ* Adiponectin, C1Q and collagen domain containing, *C/EBPα/β* CCAAT enhancer binding protein alpha/beta, *COL1A1* Collagen type I alpha 1 chain, *FABP4* Adipocyte fatty acid binding protein 4, *FASN* Fatty acid synthase, *G0S2* G0/G1 switch 2, *H-FABP* Heart-type fatty acid binding protein, *IGFBP7* Insulin-like growth factor binding protein 7, *PLIN2* Perilipin 2, *PPARG* Peroxisome proliferator activated receptor γ, *SCD* Stearoyl-CoA desaturase, *SLC27A1* (*FATP1*) Solute carrier family 27 member 1 (fatty acid transport protein 1), *STC2* Stanniocalcin 2, *SREBP1* Sterol regulatory element binding protein 1, *TIMP2* Tissue inhibitor of metalloproteinases 2, *TMEM164 *Transmembrane protein 164

^c^*AMPK* Protein kinase AMP-activated catalytic subunit alpha 1 signaling pathway, *ECM–receptor interactions* Extracellular matrix (ECM)–receptor interactions, *PPAR* Peroxisome proliferator-activated receptor, *Wnt/β-catenin* Wingless and Int (Wnt)/β-catenin signaling pathway

Nutrigenomics research has revealed that fructose enhances IMF deposition by activating ChREBP, providing new targets for nutritional interventions (Tables 4 and 8). Adjusting dietary components such as energy, protein, amino acids, fatty acids, and phytochemicals has been shown to significantly improve meat quality in broilers. In particular, supplements of ω-3 PUFAs and CLAs have notable effects on meat quality. A dietary ω-6/ω-3 PUFA ratios between 4:1 and 2.5:1 has significantly enhanced IMF deposition and meat quality [160], though their dosage must be carefully balanced to avoid adverse effects like reduced antioxidant capacity [164].

**Table 8:** Key findings in the dietary regulation of IMF in chickens

Diets^a^		Chicken breeds	IMF^b^	References
Direct intake				
15% CP with 11.51 MJ/kg ME		Beijing-you	IMF ↑	[6]
15% CP with 12.56 MJ/kg ME		Taihe Silky Fowl		[122]
168% dArg of the Ross catalog		Ross broilers		[137]
13.5% CP with 11.75 MJ/kg ME		Roman		[204]
Selenomethionine (0.3 mg/kg); *Bacillus subtilis* (10^9^ CFU/kg); or selenomethionine (0.3 mg/kg) + *Bacillus subtilis* (10^9^ CFU/kg)		Ross 308		[205]
Vitamin E (100 IU/kg)		Arbor Acres		[206]
Vitamin D_3_ (3,750 IU/kg)		Luhua		[207]
Rutin (400 mg/kg)		Qingyuan partridge		[208]
Fermented citrus pomace (10%)		Qingyuan partridge		[210]
Fresh corn extract (0.6%)		Jingyuan		[211]
Fructose (10%)		Lingshan chicken, yellow-feather chicken		[1]
18% CP (Starter)		Ross PM3	IMF ↓	[118]
Lys and Met exceeded NRC (1994) recommendation by 40%		Ross 308		[133]
2% DHA-rich microalgae		Cornish		[159]
Chinese Yam polysaccharide (250 or 500 mg/kg)		Crossbred chickens		[209]
Maternal characteristics or nutrition treatments				
Energy restriction, −2.34 MJ/kg and −3.51 MJ/kg		Arbor Acres	IMF ↑	[218]
Energy restriction 25%		Fat-line		[219]
In ovo feeding				
N-carbamylglutamate (2 mg)		Ross 308	IMF ↑	[244, 245]
* Lactobacillus plantarum* + Lupin RFO; 2 mg of raffinose family oligosaccharides, enriched with 10^5^ bacteria CFU of *Lactobacillus plantarum*		Cobb 500	IMF ↓	[256]
Galactooligosaccharides (3.5 mg) or sodium butyrate (0.6 mg)		Ross 308		[258]

^a^The values represent the content or level of addition in the diets, unless otherwise specified. *CFU* Colony forming unit, *CP* Crude protein, *DHA* Docosahexaenoic acid, *dArg* Digestible arginine, *Lys* Lysine, *Met* Methionine, *ME* Metabolizable energy, *NRC* National Research Council Nutrient Requirements of Poultry, *RFO* Raffinose family oligosaccharides

^b^*IMF* Intramuscular fat; ↑: Increased or improved compared to the control group without treatment; ↓: Decreased or diminished compared to the control group without treatment

Maternal nutrition and IOF have also been confirmed to significantly impact offspring meat quality, which opens new avenues for improving embryonic nutrition. Maternal diets supplemented with specific trace elements and vitamins have enhanced muscle development and antioxidant capacity in offspring, thereby improving meat quality [225, 228, 235]. Integrating genetic and nutritional approaches to regulate the commitment of MSCs is likely to be an effective strategy to precisely regulate IMF formation. For example, using genomic selection to identify broilers with superior genetic potential for IMF deposition. When combined with precision nutrition, such as the supplementation of rutin to inhibit AMPK signaling, this approach increases IMF deposition, thereby enhancing chicken meat flavor and texture [208].

These contributions and innovations provide new research directions for improving chicken meat quality, while offering theoretical support and technical guidance for the sustainable development of the poultry industry. It is possible to enhance meat quality while maintaining broiler growth performance, thereby driving the poultry industry towards higher quality and more sustainable development.

## Future directions

Meat quality is considered to be a complex, multidimensional issue involving genetics, nutrition, and other factors. Research into chicken meat quality traits has advanced significantly in recent decades, yet noteworthy challenges and opportunities warranting further exploration remain. Future research should focus on five key areas: the interactions between nutrients and genetic regulatory elements, the commitment of MSCs, the nutrition of the embryonic phase, the interaction between muscle and adipose tissues, and the strategies of precision nutrition. These research directions are interconnected and mutually supportive. In-depth exploration of these areas is expected to provide a comprehensive understanding of the mechanisms underlying IMF deposition in broilers at the molecular, cellular, and individual levels, which will optimize meat quality and provide novel strategies and technical support for the sustainable development of the broiler industry.

### Nutrient–genetic interactions

The interactions between nutrients and genetic regulatory elements are foundational, as they explain how specific nutrients affect key genes, proteins, ncRNAs, and epigenetic modifications, ultimately impacting meat quality. Understanding these interactions is crucial for developing nutritional strategies to enhance IMF deposition and overall meat quality. Advances in nutrigenomics have already identified genetic links between certain nutrients, such as proteins, amino acids, vitamins, phytochemicals, and other compounds, and IMF deposition in chickens, as discussed in Sect. "IMF by nutrigenomics". Future research should delve deeper into the mechanisms of action of more nutrients at the cellular and genetic levels.

### MSCs commitment

**Fig. 6:**
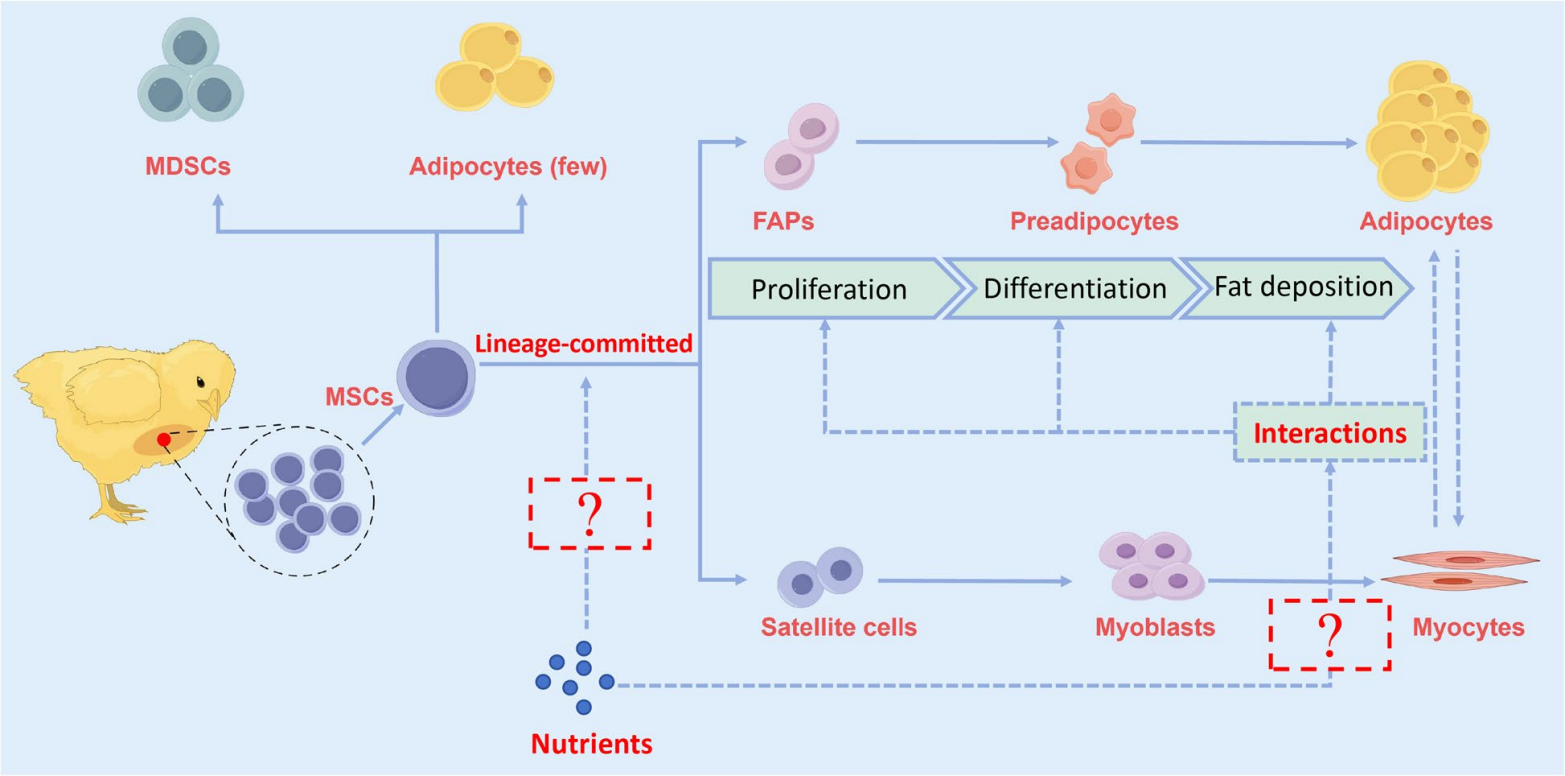
A schematic diagram for further research points in the targeted regulation of intramuscular fat deposition and improvement of meat quality. The solid arrows indicate the proliferation or differentiation of cells, and the dashed arrows represent interactions between nutrients and intercellular signaling. The red dashed rectangle and question mark indicate that these paths have not yet been fully investigated in chickens. FAPs: Fibro-adipogenic progenitors; MDSCs: Muscle-derived stem cells; MSCs: Mesenchymal stem cells. Created by FigDraw (https://www.figdraw.com)

Based on the insights into the interactions between nutrients and genetics, understanding the mechanisms that regulate the commitment of MSCs into myogenic or adipogenic lineages is a core research area. In muscle tissue, both myocytes and adipocytes originate from MSCs, as shown in Fig. 6. Identifying the ‘switches’, such as Wnt/β-catenin and lncRNA *IMFNCR*, that control these processes and elucidating their interaction mechanisms with nutrients are crucial for regulating IMF deposition and improving meat quality. This requires developing effective methods for isolating and culturing chicken FAPs and establishing FAP cell lines, which will facilitate detailed studies of these regulatory mechanisms.

### Embryonic nutrition

Embryonic nutrition has been demonstrated to determine meat quality-related muscle characteristics, including myofiber type and the number of adipocytes, particularly during critical periods such as embryonic, fetal, and neonatal development [13, 290]. This area is closely related to the commitment of MSCs, as it can shape the early developmental trajectories of myogenesis and adipogenesis. Future research should delve deeper into this area, especially the effects of embryonic fatty acid nutrition on the differentiation of MuSCs and FAPs, which would enhance strategies for improving IMF content and meat quality at individual levels post-hatch.

### Muscle–adipose interactions

The interaction between muscle and adipose tissue, mediated by cytokines, ncRNAs, and metabolites, is crucial for intramuscular fat deposition [287–289]. Myofibers and adipocytes secrete these factors via autocrine, endocrine, and paracrine pathways, forming MuSC niches that regulate muscle remodeling and adipose metabolism [291]. This area is closely related to MSCs commitment and embryonic nutrition, as both processes affect the cellular environment and signaling pathways in muscle tissue. A comprehensive investigation of this area can provide new targets for regulating IMF deposition. Exosome research has revealed novel mechanisms of intercellular communication between MuSCs and FAPs [287–289]. In chickens, research in this area remains limited [99]. Future studies should focus on the roles of cytokines, ncRNAs, and metabolites carried by exosomes in muscle–adipose tissue interactions to enrich our understanding of the mechanisms underlying meat quality regulation.

### Precision nutrition strategies

Integrating the findings from the aforementioned fields is essential for implementing precision nutrition strategies. These strategies are critical for enhancing IMF content and meat quality. They are specifically tailored to meet the unique nutrient requirements of broiler chickens at different growth stages, genders, and breeds. Implementing these strategies also entails exploring how environmental factors interact with genetic and nutritional factors to collectively determine meat quality. By integrating key targets identified in genetic research that regulate MSCs commitment and muscle‒adipose tissue interactions, the optimal timing for nutritional intervention can be determined. This enables precise regulation of skeletal muscle development and IMF deposition, ultimately leading to the improvement of chicken meat quality. For example, using genomic selection to identify broilers with superior genetic potential for IMF deposition. Combined with precision nutrition strategies, such as supplementing specific plant extracts (e.g., rutin) to inhibit the AMPK signaling pathway, which aims to increase IMF deposition and enhance chicken meat flavor and texture [208]. Supplementing CYP to suppress IMF deposition by activating the Wnt signaling pathway to promote muscle development in chickens [209]. Future research should further explore the mechanisms of genetic and nutritional synergy, such as combining gene editing technologies with precision nutrition strategies, to optimize broiler growth performance and meat quality.

Current technological advancements have laid a solid groundwork for achieving the aforementioned objectives. Single-cell omics technologies can facilitate the analysis of MSCs commitment and differentiation mechanisms and the identification of key cell subsets and molecular features that regulate IMF formation [64, 65, 292]. Multiomics integration strategies (e.g., genomics, transcriptomics, proteomics, and metabolomics) will provide a comprehensive illustration of the gene expression, protein synthesis, and metabolite profiles under nutritional intervention [293]. These strategies also aid in constructing a regulatory network for meat-quality-related traits. Gene-editing technologies such as CRISPR/Cas9 enable the precise knockout or modification of target genes, thereby verifying their roles in muscle development and fat deposition [294]. Studies such as that by Xu et al. [295], which utilize gene-editing technology to elucidate the roles of specific genes in chicken meat development, further underscore the potential of these technologies. Real-time imaging technologies dynamically monitor cell-biological processes, revealing how early nutrition influences muscle-growth trajectories [296]. The integration of these cutting-edge technologies is expected to help overcome challenges in meat quality improvement, thereby enhancing the efficiency, quality, and sustainability of poultry industry.

## Future perspectives

In practical production, enhancing chicken meat quality requires the comprehensive consideration of genetics, nutrition, and environmental factors to achieve a balance between economic benefits and meat quality. Genetic improvement is committed to selecting breeds with high IMF content, but it is essential to ensure that growth efficiency is not compromised. Nutritional intervention needs to precisely adjust feed components based on the broiler breed and growth stage to balance IMF deposition and growth performance.

For breeders, utilizing genomic selection to identify breeder chickens carrying favorable genotypes (e.g., the BB genotype at the *FABP4* gene) for reproduction [28], thereby increasing the IMF content of their offsprings. Developing molecular markers based on key genes, such as *FASN* and *LPL*, can facilitate early selection and precision breeding. For example, the rs315349829 mutation in the *FASN* gene has been discovered to be significantly associated with IMF content, and chickens carrying this mutation exhibit an average increase of 15% in IMF [65]. Marker-assisted breeding can screen out chicks with high IMF potential at an early stage, thereby shortening the breeding cycle.

For farmers, enhancing the IMF content of chickens while maintaining good growth performance requires optimizing feed formulations and management practices. This includes adjusting the dietary proportions of energy, protein, amino acids, vitamins, and fatty acids. For instance, increasing the contents of vitamins, such as vitamin E or vitamin D_3_ has been found to boost IMF deposition [206, 207], but the dosage must be balanced to prevent negative impacts on growth performance. Supplementing with specific amino acids, such as Met and Lys, can help maintain the growth rate of chickens. Supplementing with exogenous phytochemicals, like rutin, fermented citrus pomace, and fresh corn extract, have also been confirmed to increase IMF [208, 210, 211]. Appropriately adjusting the nutritional intake of breeder chickens can optimize the IMF content of their offsprings, thereby preventing potential declines in production performance and metabolic disorders induced by overnutrition. In terms of environmental management, optimizing the rearing conditions is necessary to maximize the growth and meat quality potential of broilers. For example, ensuring proper ventilation and maintaining an appropriate temperature in the poultry house can reduce stress responses, which in turn improves the health and growth performance of chickens.

For the poultry industry, it is necessary to establish standards to promote the production of high-quality chicken meat, support the research and development as well as the promotion of advanced technologies, and strengthen the coordination of the industrial chain to achieve sustainable development. Developing feed additives based on PPARγ agonists, which possess the potential to regulate adipose metabolism and enhance IMF content in chickens, is a promising approach. Utilizing gene-editing technology to precisely regulate key genes (such as *FASN*) to develop high-quality chicken breeds with high IMF traits is also a potential strategy. When applying nutritional regulation strategies, it is necessary to weigh the economic benefits brought by the increased IMF content against the cost inputs. Specific nutrients, like rutin and fresh corn extract, may increase feed costs, but they can enhance the quality and market value of chicken meat. Increasing IMF content may affect production performance. High-energy diets have been found to promote IMF deposition, but they may also increase the metabolic burden on broilers, leading to excessive accumulation of abdominal fat. Excessive intake of PUFAs may slow down growth rates. Therefore, it is necessary to conduct a comprehensive assessment of the cost–benefit ratio, reduce costs through precision nutrition strategies, and develop low-cost functional feeds derived from by-products.

Future research needs to delve into the mechanisms of the synergistic effects between genetics and nutrition, the directed differentiation mechanisms of MSCs, and the regulation of muscle development by embryonic nutrition. It also needs to enhance the study of the interaction between muscle and adipose tissues, implement precise nutritional strategies, and integrate multiomics technologies to elucidate the mechanisms of meat quality regulation. These efforts will provide more scientific guidance for poultry farming and help the poultry industry develop towards high quality and sustainability.

## Supplementary Information

Supplementary Material 1. Annotations of key genes or proteins in Table 1.

Supplementary Material 2. Annotations of key genes or proteins in Table 4.

Supplementary Material 3. Annotations of Fig. 5.

## Data Availability

Not applicable.

## Abbreviations

AAs: Amino acids
ALA: Alpha-linolenic acid
AMEn: Apparent metabolizable energy
BCAAs: Branched-chain amino acids
CLAs: Conjugated linoleic acids
circRNAs: Circular RNAs
DEGs: Differentially expressed genes
DHA: Docosahexaenoic acid
DMGs: Differentially methylated genes
DNL: De novo lipogenesis
dArg: Digestible arginine
dLys: Digestible lysine
dThr: Digestible threonine
EPA: Eicosapentaenoic acid
FAPs: Fibro-adipogenic progenitors
Gly: Glycine
Ile: Isoleucine
IMF: Intramuscular fat
IOF: In ovo feeding
LC-PUFAs: Long-chain polyunsaturated fatty acids
Leu: Leucine
lncRNAs: Long noncoding RNAs
ME: Metabolizable energy
Met: Methionine
miRNAs: MicroRNAs
MSCs: Mesenchymal stem cells
MuSCs: Muscle stem cells
ncRNAs: Noncoding RNAs
PUFAs: Polyunsaturated fatty acids
RFI: Residual feed intake
RME_m_: Residual maintenance requirement
SNPs: Single nucleotide polymorphisms
TG: Triglycerides
Trp: Tryptophan
Val: Valine
WHC: Water-holding capacity
ω-3: Omega-3
ω-6: Omega-6
