# Supplementary material for: Research advances in intramuscular fat deposition and chicken meat quality: genetics and nutrition
Source: J Anim Sci Biotechnol. 2025 Jul 16;16:100. doi: 10.1186/s40104-025-01234-5 (PMC12265352; doi:10.1186/s40104-025-01234-5)
Supplement: Supplementary file 1 — Supplementary Material 1. Annotations of key genes or proteins in Table 1. [file 40104_2025_1234_MOESM1_ESM.docx]

Annotations of key genes or proteins in Table 1

*ABCA1*: ATP binding cassette subfamily A member 1;

*ACAD9*: Acyl-CoA dehydrogenase family member 9;

*ACADL*: Acyl-CoA dehydrogenase, long chain;

*ACAT1*: Acetyl-CoA acetyltransferase 1;

*ACOT9*: Acyl-CoA thioesterase 9;

*ACOX2*: Acyl-CoA oxidase 2;

*ACOX3*: Acyl-CoA oxidase 3, pristanoyl;

*ACSBG1*: Acyl-CoA synthetase bubblegum family member 1;

*ACSL1*: [Acyl-CoA synthetase long chain family member 1](https://www.ncbi.nlm.nih.gov/gene/2180);

*ADIPOQ*: Adiponectin, C1Q and collagen domain containing;

*ANGPTL4*: Angiopoietin-like 4;

*APOA1*: Apolipoprotein A1;

APOC3: [Apolipoprotein C3](https://www.ncbi.nlm.nih.gov/gene/345);

*APOO*: Apolipoprotein O;

*ATP5B*: ATP synthase beta chain, mitochondrial;

AUP1: CUE domain-containing protein;

*BCAT1*: Branched chain amino acid transaminase 1;

*B4GALT3*: Beta-1,4-galactosyltransferase 3;

*BEND6*: BEN domain containing 6;

*BMPR2*: Bone morphogenetic protein receptor 2;

*BOP1*: Block of proliferation 1;

*BRCA1*: [BRCA1 DNA repair associated](https://www.ncbi.nlm.nih.gov/gene/672);

*CAPN2*: Calpain 2;

CASP3: Apoptosis-inducing enzyme caspase-3;

CRAT: Carn-acyltransf domain-containing protein;

*CD36*: Cluster of differentiation 36;

*CDK1*: [Cyclin dependent kinase 1](https://www.ncbi.nlm.nih.gov/gene/983);

*C/EBPα/β*: CCAAT enhancer binding protein alpha/beta;

*CETP*: Cholesteryl ester transfer protein;

*CH25H*: Cholesterol 25-hydroxylase;

*CIDEC*: Cell death-inducing DNA fragmentation factor-like effector C;

*COL1A1*: Collagen type I alpha 1 chain;

*COL1A2*: Collagen type I alpha 2 chain;

COL4A1: [Collagen type IV alpha 1 chain](https://www.ncbi.nlm.nih.gov/gene/1282);

*COL6A1*: Collagen type VI alpha 1 chain;

*COL6A2*: Collagen type VI alpha 2 chain;

*COL6A3*: Collagen type VI alpha 3 chain;

*CPT1A*: Carnitine palmitoyl transferase 1A;

*CPT2*: Carnitine palmitoyltransferase II;

*CUBN*: Cubilin;

*CYP1B1*: Cytochrome P450 family 1 subfamily B member 1;

*DAK*: Dihydroxyacetone kinase;

*DGAT2*: Diacylglycerol o-acyltransferase 2;

*DHCR24*: 24-dehydrocholesterol reductase;

*DHCR7*: [7-dehydrocholesterol reductase](https://www.ncbi.nlm.nih.gov/gene/1717);

*DHDH*: [Dihydrodiol dehydrogenase](https://www.ncbi.nlm.nih.gov/gene/27294);

*DNAJC27*: [DnaJ heat shock protein family (Hsp40) member C27](https://www.ncbi.nlm.nih.gov/gene/51277);

*ECH1*: Enoyl-CoA hydratase 1;

*EDN2*: Endothelin 2;

*EHHADH*: Enoyl-CoA hydratase and 3-hydroxyacyl CoA dehydrogenase;

*ENSGALG00000041996*: An unannotated lncRNA candidate identified through RNA-Seq analysis, which may regulate the expression of CD36 and ACADL;

*FABP4*: Adipocyte fatty acid binding protein 4;

*FABP5*: Fatty acid binding protein 5;

*FATP4*: Fatty acid transport protein 4;

*FASN*: Fatty acid synthase;

*FBP1*: Fructose-bisphosphatase 1;

*FKBP1B*: [FKBP prolyl isomerase 1B](https://www.ncbi.nlm.nih.gov/gene/2281);

*FUT9*: Fucosyltransferase 9;

*G0S2*: G0/G1 switch 2;

*G3BP1*: [G3BP stress granule assembly factor 1](https://www.ncbi.nlm.nih.gov/gene/10146);

*GAPDH*: Glyceraldehyde-3-phosphate dehydrogenase;

*GBE1*: 1,4-alpha-glucan branching enzyme 1;

*GCNT1*: Glucosaminyl (N-acetyl) transferase 1;

*GPX1*: Glutathione peroxidase 1;

*GSTT1L*: Glutathione S-transferase theta 1-like;

*HADHA*: Hydroxyacyl-CoA dehydrogenase/3-ketoacyl-CoA thiolase/enoyl-CoA hydratase (trifunctional protein), alpha subunit;

*HADHB*: Hydroxyacyl-CoA dehydrogenase/3-ketoacyl-CoA thiolase/enoyl-CoA hydratase (trifunctional protein), beta subunit;

*HAT1*: Histone acetyltransferase 1;

*H-FABP* (*FABP3*): Heart-type fatty acid binding protein (fatty acid binding protein 3);

*HIBCH*: 3-hydroxyisobutyryl-CoA hydrolase;

*HMGCLL1*: 3-hydroxymethyl-3-methylglutaryl-CoA lyase like 1;

*HMGCR*: 3-hydroxy-3-methylglutaryl-CoA reductase;

*HPGD*: 15-hydroxyprostaglandin dehydrogenase;

*HSPB1*: Heat shock protein family B (small) member 1;

*L3MBTL1*: L3MBTL histone methyl-lysine binding protein 1;

*IGFBP7*: Insulin-like growth factor binding protein 7;

LAMB4: [Laminin subunit beta 4](https://www.ncbi.nlm.nih.gov/gene/22798);

*LDHA*: Lactate dehydrogenase A;

*LMOD2*: Leiomodin 2;

*LPIN1*: Lipin 1;

*LPL*: Lipoprotein lipase;

*LSS*: Lanosterol synthase;

*LY86*: Lymphocyte antigen 86;

*MEGF10*: Multiple EGF-like domain 10;

MRC2: C-type mannose receptor 2;

*MSMO1*: Methylsterol monooxygenase 1;

*NDUFA9*: [NADH:ubiquinone oxidoreductase subunit A9](https://www.ncbi.nlm.nih.gov/gene/4704);

*NDUFAB1*: NADH:ubiquinone oxidoreductase subunit AB1;

*NSDHL*: NAD (P)-dependent steroid dehydrogenase-like;

PAK2 : [p21 (RAC1) activated kinase 2](https://www.ncbi.nlm.nih.gov/gene/5062);

*PCCB*: Propionyl-CoA carboxylase beta subunit;

*PEMT*: [Phosphatidylethanolamine N-methyltransferase](https://www.ncbi.nlm.nih.gov/gene/10400);

*PHKA1*: Phosphorylase kinase regulatory subunit alpha 1;

*PLA2G4F*: Phospholipase A2 group IVF;

*PIGO*: [Phosphatidylinositol glycan anchor biosynthesis class O](https://www.ncbi.nlm.nih.gov/gene/84720);

*PLIN1*: Perilipin 1;

*PLIN2*: Perilipin 2;

*PNPLA2*: Patatin-like phospholipase domain containing 2;

*PPARG*: Peroxisome proliferator activated receptor γ;

*PPARGC1A/PGC-1α*: Peroxisome proliferator-activated receptor gamma coactivator 1-alpha;

*PRKAG2*: [Protein kinase AMP-activated non-catalytic subunit gamma 2](https://www.ncbi.nlm.nih.gov/gene/51422);

*RBP7*: Retinol binding protein 7;

*RPS3*: [Ribosomal protein S3](https://www.ncbi.nlm.nih.gov/gene/6188);

*RPS6KB1*: [Ribosomal protein S6 kinase B1](https://www.ncbi.nlm.nih.gov/gene/6198);

*SCARA5*: [Scavenger receptor class A member 5](https://www.ncbi.nlm.nih.gov/gene/286133);

*SCD*: Stearoyl-CoA desaturase;

*SCT2*: Stanniocalcin 2;

*SLC16A7*: Solute carrier family 16 member 7 gene;

*SLC27A1* (*FATP1*): Solute carrier family 27 member 1 (fatty acid transport protein 1);

SLC7A5: large neutral amino acids transporter small subunit 1;

*SLC1A6*: Solute carrier family 1 member 6;

*SLC45A3*: Solute carrier family 45 member 3;

*SNAI2*: Snail family transcriptional repressor 2;

SNPs: Single nucleotide polymorphisms;

*SNX4*: Sorting nexin 4;

*SREBP1*: Sterol regulatory element binding protein 1;

*ST8SIA5*: ST8 alpha-N-acetyl-neuraminide alpha-2,8-sialyltransferase 5;

STMN1: [Stathmin 1](https://www.ncbi.nlm.nih.gov/gene/3925);

*TECRL*: Trans-2,3-enoyl-CoA reductase like;

TGF-β: Transforming growth factor-beta;

*THBS1*: Thrombospondin 1;

*THRSP*: Thyroid-hormone responsive protein;

TIMP2: Tissue inhibitor of metalloproteinases 2;

*TMEM164*: Transmembrane protein 164;

*TMEM38B*: [transmembrane protein 38B](https://www.ncbi.nlm.nih.gov/gene/55151);

*TNIP1*: TNFAIP3 interacting protein 1;

*UCP3*: Uncoupling protein 3;

*ZNF488*: [Zinc finger protein 488](https://www.ncbi.nlm.nih.gov/gene/118738).
