# Supplementary material for: Research advances in intramuscular fat deposition and chicken meat quality: genetics and nutrition
Source: J Anim Sci Biotechnol. 2025 Jul 16;16:100. doi: 10.1186/s40104-025-01234-5 (PMC12265352; doi:10.1186/s40104-025-01234-5)
Supplement: Supplementary file 2 — Supplementary Material 2. Annotations of key genes or proteins in Table 4. [file 40104_2025_1234_MOESM2_ESM.docx]

Annotations of key genes or proteins in Table 4

*ACACA*: Acetyl-CoA carboxylase alpha;

*ACOX2*: [Acyl-CoA oxidase 2](https://www.ncbi.nlm.nih.gov/gene/8309);

*ADIPOQ*: [Adiponectin, C1Q and collagen domain containing](https://www.ncbi.nlm.nih.gov/gene/9370);

*AGTR1*: Angiotensin II receptor type 1;

*AMPKα*: AMP-activated protein kinase alpha subunit;

*APOA1*: [Apolipoprotein A1](https://www.ncbi.nlm.nih.gov/gene/335);

*ASB2*: Ankyrin repeat and SOCS box containing 2;

*β-catenin*: Catenin beta 1;

*C/EBPα*: CCAAT enhancer binding protein alpha;

*C/EBPβ*: CCAAT/enhancer binding protein beta;

*CAPN2*: Calpain 2;

*CAPN3*: Calpain 3;

*CD36*: Cluster of differentiation 36;

*CERS1*: Ceramide synthase 1;

*CERS6*: [Ceramide synthase 6](https://www.ncbi.nlm.nih.gov/gene/253782);

*CETP*: Cholesteryl ester transfer protein;

ChREBP: MLX interacting protein-like;

*CYR61*: Cysteine-rich angiogenic inducer 61;

*ELOVL7*: ELOVL fatty acid elongase 7;

*FABP4*: Adipocyte fatty acid binding protein 4;

*FABP5*: [Fatty acid binding protein 5](https://www.ncbi.nlm.nih.gov/gene/2171);

*FADS1*: Fatty acid desaturase 1;

*FASN*: Fatty acid synthase;

*FAT*: Fatty acid translocase;

*FATP1*: Fatty acid transport protein 1;

*FM*: Fast myosin heavy chain;

*GLB1L*: Galactosidase beta 1 like;

*H-FABP*: Heart-type fatty acid binding protein;

*HPGD*: 15-hydroxyprostaglandin dehydrogenase;

*IGF-1*: Insulin-like growth factor 1;

*LPL*: Lipoprotein lipase;

*Myf5*: Myogenic factor 5;

*MyoG*: Myogenin;

*MyoD*: Myoblast determination factor;

*PDK4*: Pyruvate dehydrogenase kinase 4;

*PLIN2*: Perilipin 2;

*PPARG*: Peroxisome proliferator activated receptor γ;

*SGMS2*: Sphingomyelin synthase 2;

*SLC27A1* (*FATP1*): Solute carrier family 27 member 1 (fatty acid transport protein 1);

*SM*: Slow myosin heavy chain;

*S1PR3*: [Sphingosine-1-phosphate receptor 3](https://www.ncbi.nlm.nih.gov/gene/1903);

*SPHK1*: Sphingosine kinase 1;

*SREBP1*: Sterol regulatory element binding protein 1;

*UCP3*: Uncoupling protein 3;

*UGCG*: UDP-glucose ceramide glucosyltransferase;

*UGT8*: UDP glycosyltransferase 8;

*Wnt1*: Wnt family member 1.
