# Supplementary material for: Research advances in intramuscular fat deposition and chicken meat quality: genetics and nutrition
Source: J Anim Sci Biotechnol. 2025 Jul 16;16:100. doi: 10.1186/s40104-025-01234-5 (PMC12265352; doi:10.1186/s40104-025-01234-5)
Supplement: Supplementary file 3 — Supplementary Material 3. Annotations of Fig. 5. [file 40104_2025_1234_MOESM3_ESM.docx]

Annotations of Fig. 5

1—Myogenesis and adipogenesis, from left to right, represent the process of mesenchymal cells (MSCs) differentiating into myocytes and mature adipocytes. This process involves the initial regulation of pathways such as Wnt/β-catenin, AMPK, and SHH signaling. Blue dashed arrows indicate that maternal nutrition or in ovo feeding (IOF) may affect the commitment or determination of chicken MSCs.

2—The three sub-figures from left to right represent the treatments of maternal nutrition, IOF, and direct uptake, with the green arrows indicating the corresponding order that matches these figures.

3—Fatty acids released from very-low-density lipoproteins (VLDL) can penetrate myocytes and adipocytes, where they are re-synthesized into triglycerides (TG) and deposited. The fat accumulated in chicken muscle tissues can originate from either dietary fats (portomicrons; circle 3) or plasma VLDL-mediated transport (circle 5).

4—Nutrients and bioactives, along with their metabolites, promote the adipogenic differentiation of intramuscular preadipocytes via direct uptaking by regulating the expression of peroxisome proliferator-activated receptor gamma (*PPARG*), such as fructose, rutin, and vitamins E and D_3_ [1, 206-208].

6—The crucial role of exogenous fatty acids (from VLDL or diet) in regulating *PPARG* expression and adipogenesis.

7—Single-cell RNA sequencing confirmed that de novo lipogenesis (DNL) mainly occurs in myocytes, contributing to intramuscular fat (IMF) deposition. This process is regulated by multiple genes, such as acetyl-CoA acyltransferase 2 (*ACAA2*), hydroxyacyl-CoA dehydrogenase trifunctional multienzyme complex subunit alpha (*HADHA*), hydroxyacyl-CoA dehydrogenase (*HADH*), fatty acid synthase (*FASN*), and hydroxyacyl-thioester dehydratase type 2 (*HTD2*).

8—The red dashed box indicates non-coding RNAs that have regulatory effects on adipogenesis-related genes. The pink boxes reveal the sequence of transcription factors controlling adipogenesis. The early transcriptional regulators, including CCAAT/enhancer binding-protein (C/EBPβ and C/EBPδ) are expressed first and subsequently stimulate the key transcription factors, including PPARγ and C/EBPα. At the terminal differentiation stage, PPARγ and C/EBPα regulate the transcription of various adipocyte-associated genes, such as lipoprotein lipase (*LPL*), fatty-acid-binding proteins (*FABPs*), cluster of differentiation 36 (*CD36*), and adiponectin, C1Q and collagen domain-containing (*ADIPOQ*). PPARγ and C/EBPα are the master regulators of adipogenesis and can cross-regulate each other. Sterol response element-binding protein (SREBP1) is regulated by insulin and can promote adipogenesis by enhancing the expression of *PPARG*. Kruppel-like transcription factor (KLF5) is activated by C/EBPβ/δ during the early stages of adipogenesis and subsequently promotes the expression of *PPARG*. KLF2 and GATA binding protein 2 (GATA2) are anti-adipogenic transcription factors that repress adipogenesis by inhibiting *PPARG* expression. Zinc finger protein 423 (Zfp423) regulates preadipocyte cell determination by modulating *PPARG* expression. Retinoid X receptor γ (RXRG) forms heterodimers with PPARγ, thereby enhancing the transcriptional activation of target genes. Carnitine palmitoyl transferase 1A (CPT1A), which is involved in fatty acid oxidation, interacts with PPARγ through complex regulatory mechanisms to collectively influence energy balance and lipid homeostasis. Acetyl-CoA acyltransferase 1 (ACAA1) catalyzes the terminal reaction of fatty acid β-oxidation, and its expression may be regulated by the PPAR family. GATA binding protein 6 (*GATA6*) can be upregulated post-transcriptionally through the action of *LncHLFF*, which functions as a molecular sponge for miR-2188-3p, thereby enhancing hepatic lipid synthesis.

9—The potential network of signaling pathways contributing to chicken intramuscular lipid metabolism. The pathways highlighted in gray boxes may be crucial for IMF deposition in chicken breast muscle. Activation of PPARγ in the PPAR signaling pathway upregulates lipogenic genes such as *ADIPOQ*, *LPL*, *SCD*, and *CD36*, thereby promoting TG synthesis.

10—PPARγ may enhance the interaction between PLIN1 and CIDEC, accelerating IMF deposition. In the steroid biosynthesis pathway, upregulation of cholesterol synthesis genes like *DHCR24* and *NSDHL* may increase steroid ester synthesis. Abbreviations: AMPK: Protein kinase AMP-activated catalytic subunit alpha 1 signaling pathway; *APOA1*: Apolipoprotein A1; *CH25H*, Cholesterol 25-hydroxylase; CIDEC: Cell death-inducing DNA fragmentation factor-like effector C; *COL1A1*: Collagen type I alpha 1 chain; *DHCR24*: 24-Dehydrocholesterol reductase; ECM‒receptor interactions: Extracellular matrix receptor interactions; FAPs: Fibro-adipogenic progenitors; G3P: Glycerol-3-Phosphate; *LSS*: Lanosterol synthase; *MSMO1*: Methylsterol monooxygenase 1; *NSDHL*: NAD(P) dependent steroid dehydrogenase-like; PPAR/PPARγ: Peroxisome proliferator-activated receptor (PPAR) and its subtype PPARγ; PLIN 1: Perilipin 1; *SCD*: Stearoyl-CoA desaturase; SEs: Sterol esters; SHH: Sonic hedgehog signaling pathway; Wnt/β-catenin: Wingless and Int (Wnt)/β-catenin signaling pathway.
